# Pyrazines from bacteria and ants: convergent chemistry within an ecological niche

**DOI:** 10.1038/s41598-018-20953-6

**Published:** 2018-02-07

**Authors:** Eduardo A. Silva-Junior, Antonio C. Ruzzini, Camila R. Paludo, Fabio S. Nascimento, Cameron R. Currie, Jon Clardy, Mônica T. Pupo

**Affiliations:** 1 Faculdade de Ciências Farmacêuticas de Ribeirão Preto, Universidade de São Paulo, Ribeirão Preto, SP, 14040903 Brazil; 2 Department of Biological Chemistry and Molecular Pharmacology, Harvard Medical School, Boston, MA 02115 United States of America; 3 Departamento de Biologia, Faculdade de Filosofia, Ciências e Letras de Ribeirão Preto, Universidade de São Paulo, Ribeirão Preto, SP, 14040901 Brazil; 4 Department of Bacteriology, University of Wisconsin, Madison, WI 53706 United States of America; 5 Present Address: Department of Veterinary Microbiology, University of Saskatchewan, Saskatoon, SK S7N 5B4 Canada

## Abstract

Ants use pheromones to coordinate their communal activity. Volatile pyrazines, for instance, mediate food resource gathering and alarm behaviors in different ant species. Here we report that leaf-cutter ant-associated bacteria produce a family of pyrazines that includes members previously identified as ant trail and alarm pheromones. We found that L-threonine induces the bacterial production of the trail pheromone pyrazines, which are common for the host leaf-cutter ants. Isotope feeding experiments revealed that L-threonine along with sodium acetate were the biosynthetic precursors of these natural products and a biosynthetic pathway was proposed.

## Introduction

Leaf-cutter ants are major herbivores in the New World Tropics where they provide essential ecosystem services^1^. These ants collect fresh plant material to cultivate a symbiotic fungus, *Leucoagaricus gongylophorous*, that serves as their food source^2^. The scale at which these ants collect leaves makes them major agricultural pests in temperate regions of the New World^3,4^. Colonies of leaf-cutter ants in the genus *Atta* can have populations in the millions^5^ housed in subterranean nests that include an extensive network of tunnels and chambers which contain their fungal gardens^3^. To accomplish the task of keeping such massive populations fed, the leaf-cutter ants coordinate their harvesting behavior by using trail pheromones that are secreted from specialized poison glands^6–8^. In the case of *Atta sexdens*^8,9^, 2,5-dimethylpyrazine (**1**) and 3-ethyl-2,5-dimethylpyrazine (**2**) have been identified both as components of poison gland secretions and trail pheromones.

Pyrazines are heterocyclic natural products commonly used by ants to communicate. Mandibular glands secretions from Ponerini, Odontomachini and Ectatommini ants contain a mixture of 3-alkyl-2,5-dimethylpyrazines and 3-alkyl-2,6-dimethylpyrazines, compounds that mediate alarm behavior in *Odontomachus* workers^10,11^. Pyrazines are also components of the trail pheromones produced by the poison glands of the leaf-cutter ants *Acromyrmex octospinosus*, *Atta bisphaerica*, *Atta cephalotes*, and *Atta sexdens*^7,12,13^. These natural products likewise mediate communication in bacteria: 3,5-dimethylapyrazin-2-ol is a quorum sensing regulator implicated in biofilm formation by *Vibrio cholerae*^14^.

The bacterial associates of ants in the tribe Attine have recently been exploited as a promising source of antibiotics^15–17^ thought to protect their hosts. The production of volatiles – a critical class of molecules that guides ant behavior – by these bacteria, however, remain largely unknown. There is some evidence that volatile organic compounds (VOCs) produced by bacterial insect symbionts are sources of pheromones utilized by their hosts^18,19^. Herein, we provide the first report of a leaf-cutter ant-associated bacterium, *Serratia marcescens* 3B2, that is capable of producing pyrazines previously identified as trail pheromones (**1** and **2**) of the host ant *Atta sexdens rubropilosa* and alarm pheromones (**4** and **5**) of other ants^8,9,11,20^. The biosynthetic precursors of the trail pheromone pyrazines were unveiled by isotope feeding experiments.

## Results

### Ant associated bacteria produce a small family of pyrazines

Eight bacteria with similar morphology were isolated from *A. sexdens rubropilosa* worker ants belonging to two different colonies. The isolates were derived from two sources: whole ant bodies that had been washed with a sterile aqueous solution^21,22^ or worker ants that had been sectioned after a surface sterilization^23^. The VOCs from the eight isolates grown on agar ISP-2 medium were extracted using a SPME polydimethylsiloxane fiber and analyzed by GC-MS (Figs S1–S5, ESI^†^).

**Figure 1:**
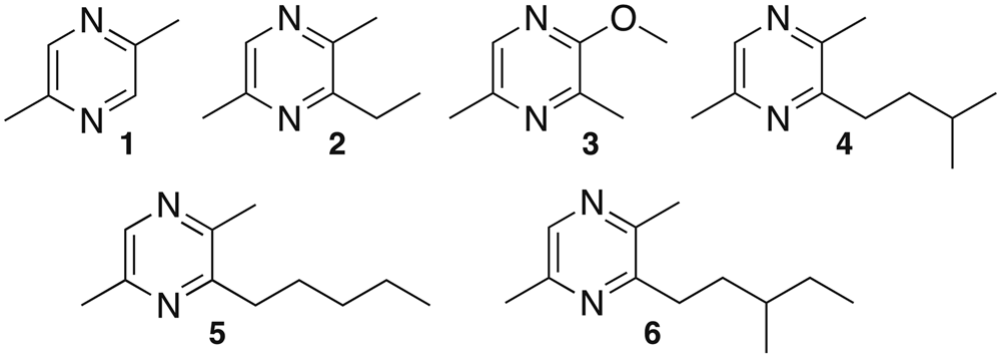
Pyrazines produced by *Serratia marcescens* 3B2.

**Figure 2:**
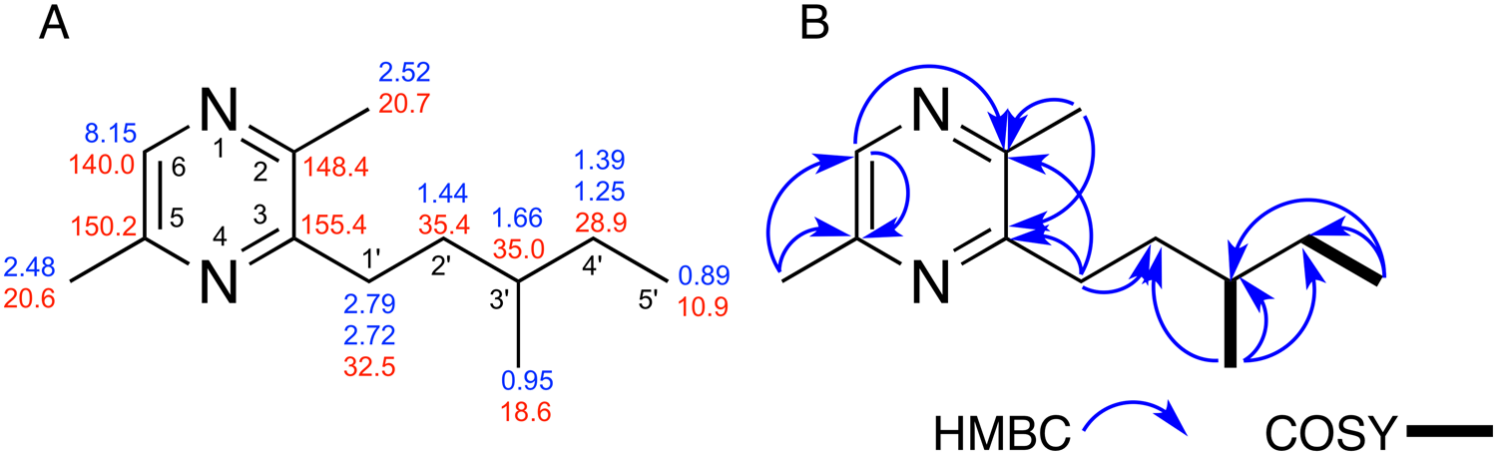
Structure, ^13^C (red) and ^1^H (blue) NMR data of compound **6** in (**A**). Key HMBC and COSY correlations (**B**).

The main compounds produced by the isolates were pyrazines **3**–**6** (Fig. 1). Compound **3** is a methoxylated analogue of 3,5-dimethylpyrazin-2-ol, a recently identified bacterial quorum sensing regulator^14^. Compounds **4** and **5** were previously reported as alarm pheromones of non-fungus growing ants^10,11^. Compound **6** (Fig. 2) is an unreported pyrazine and its structure was determined based on HRESIMS (C_12_H_20_N_2_, *m/z* 193.1694 [M + H]^+^; Fig. S10, ESI^†^) and NMR data (Table S1, ESI^†^). More specifically, the 1D and 2D NMR spectra indicated that the structure is 2,5-dimethyl-3-(3′-methylpentyl)pyrazine (Figs S6–S9, ESI^†^). *g*HMBC correlations and a key *g*COSY coupling between the methyl hydrogens (*δ* 0.95) with the hydrogen at *δ* 1.66 (H3′) established the position of this methyl group at C3′. The GC-MS fragmentation profile of **6** corroborates with NMR data, indicating a methyl group at C3′ (Fig. S3, ESI^†^).

### L-threonine induces the bacterial production of trail pheromone pyrazines

**Figure 3:**
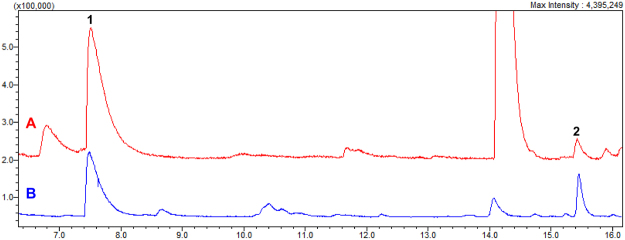
GC-MS chromatograms showing the production of the trail pheromone pyrazines **1** and **2** by *S. marcescens* 3B2 cultivated on M9 agar medium supplemented with 0.2% of glucose and 2% of L-threonine in (**A**) and their presence in gasters of *Atta sexdens rubropilosa* in (**B**).

In order to investigate if the trail pheromone pyrazines **1** and **2**, found in the hosts ants (Figs S11–S13, ESI^†^), could also be produced by the bacterium, we examined the metabolites from a single isolate – *S. marcescens* 3B2 – using minimal and defined growth media. This strain was identified by sequencing the 16S rRNA gene using the universal primers 27F and 1492R, and the resulting sequence was compared with GenBank sequences. We cultured *S. marcescens* 3B2 using M9 agar supplemented with glucose and a single amino acid: L-alanine, L-serine, L-threonine or L-valine, (Figs S14–S16, ESI^†^). Importantly, filtered sterilized amino acids were added to autoclaved media to avoid heat-induced pyrazine formation^24^. Compounds **1** and **2** were observed only in the M9 agar supplemented with glucose and L-threonine, and could also be detected in bulk extraction of the host, *Atta sexdens rubropilosa* (Fig. 3). The bacterial production of **1** and **2** was dependent on the concentration of L-threonine in the culture media, tested at 1.0, 1.5 and 2.0% (w/v; Fig. S17–S20, ESI^†^). We did not detect **1** and **2** from cultures with low L-threonine concentrations (0–0.5% w/v; Figs S17–S20, ESI^†^). In addition, L-threonine supplementation resulted in reduced yields of the more abundant pyrazines **4**–**6**, and the production of **3** was also upregulated (Fig. S17, ESI^†^).

### L-threonine and acetate are the biosynthetic precursors of the trail pheromone pyrazines

**Figure 4:**
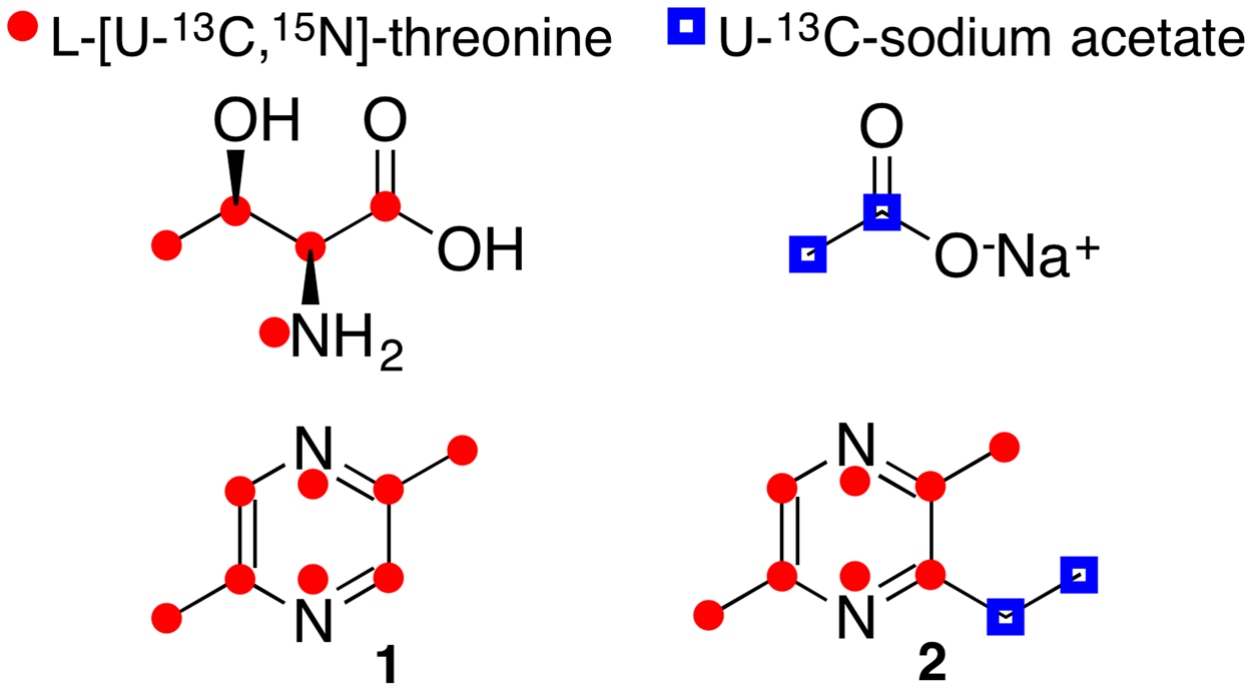
Labelling patterns of pyrazines **1** and **2** obtained after feeding the biosynthetic precursors L-[U-^13^C,^15^N]-threonine and U-^13^C-sodium acetate in *S. marcescens* 3B2 culture.

To study the role of L-threonine in the production of **1** and **2**, we grew *S. marcescens* 3B2 on M9 agar supplemented with 0.2% of glucose, 0.5% of L-alanine and 1.5% of L-threonine or L-[U-^13^C,^15^N]-threonine (Figs S21–S30, ESI^†^). GC-MS analysis showed the incorporation of eight mass units into both **1** and **2** (Figs S27 and S28, ESI^†^). The ethyl group of **2** is eliminated during GC-MS analysis and produces a fragment ion with the charge at the 2,5-dimethylpirazine ring (Fig. S4, ESI^†^). The fragment ion of **2**, produced in the medium supplemented with L-[U-^13^C,^15^N]-threonine, has the expected M + 8 shift (*m/z* 116) compared to the control (*m/z* 108), showing that the incorporation occurred just in the pyrazine ring. High-resolution electrospray ionization mass spectrometry (HR-ESIMS) analysis corroborated with the GC-MS data. The major ions observed for both **1** and **2** were consistent with the incorporation of six ^13^C and two ^15^N atoms into each (Fig. 4; Figs S29 and S30; Tables S2 and S3, ESI^†^). Minor fermentation products contained three ^13^C atoms and a single ^15^N atom, suggesting that endogenously produced L-threonine was also incorporated during the feeding experiment.

To investigate the origin of the ethyl-substitution in **2**, we added U-^13^C-sodium acetate to the growth medium. Under these conditions, no incorporation was observed into **1** whereas two additional mass units were incorporated into **2**, as evidenced by GC-MS and HR-ESI-MS (Figs S31–S35, ESI^†^). To better establish the positions of ^13^C acetate-derived atoms we took advantage of the elimination process that occurs during GC-MS of **2**. Equivalent fragment ions (*m/z* 108) were detected in both labelled (molecular ion with *m/z* 138) and control sample (molecular ion with *m/z* 136), indicating that acetate was incorporated into the eliminated ethyl group. *Serratia marcescens* 3B2 was also cultivated with ^15^N-L-alanine and 3-^13^C-L-alanine; however, no significant isotopic labelling of the 3,5-dimethylpyrazine rings of compounds **1** and **2** were observed from these amino acids (Figs S36–S45, ESI^†^). Instead, we observed low levels of a single ^13^C incorporation into the ethyl group of compound **2** when the media was supplemented with 3-^13^C-L-alanine, which is most likely due to the metabolism of L-alanine to acetate^25^. Much higher levels of M + 2 ions were observed using uniformly ^13^C labelled acetate alone or in combination with an equimolar concentration of unlabeled L-alanine (Table S3, Figs S46 and S47, ESI^†^). Altogether, the results demonstrate that acetate is the precursor of the compound **2** ethyl group. In contrast of **1** and **2**, the 2,5-dimethylpyrazine rings of **4**, **5** and **6** were labelled with ^15^N-L-alanine and 3-^13^C-L-alanine (Figs S48–S50, ESI^†^). As reported for 3,5-dimethylpyrazin-2-ol^14^, isotope labelled L-alanine and L-threonine were incorporated into the 3,5-dimethylpyrazine ring of **3** (Fig. S51, ESI^†^). These results indicate that *S. marcescens* 3B2 produces the same structural ring using different biosynthetic precursors.

**Figure 5:**
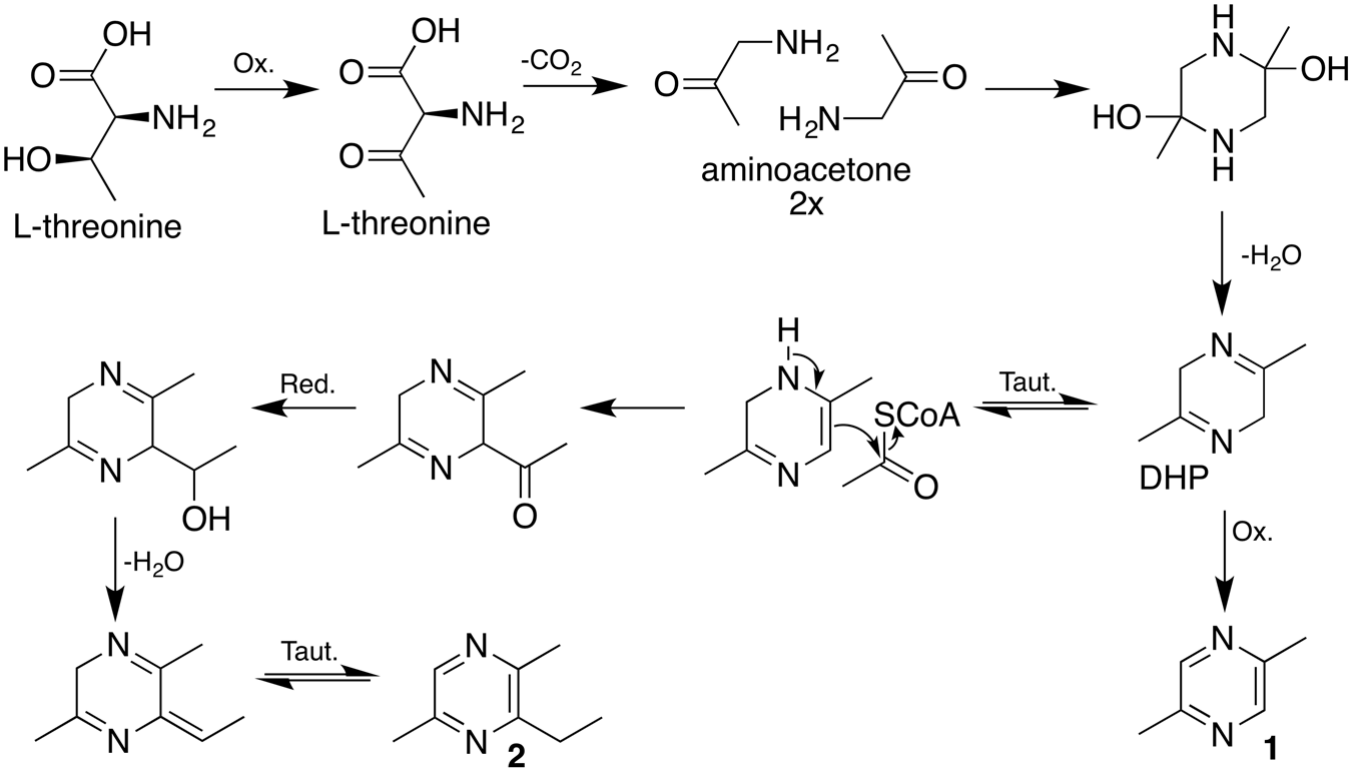
Proposed biosynthesis of pyrazines **1** and **2**, involving oxidations (ox.), reductions (red.), spontaneous tautomerization (taut.) and dehydration (−H_2_O).

We proposed the *S. marcescens* 3B2 biosynthetic pathways of **1** and **2** starting with L-threonine, which is converted to aminoacetone (Fig. 5). Condensation of two units of aminoacetone, followed by dehydration, yields the 2,5-dimethyl-3,6-dihydropyrazine (DHP), which is aromatized by ring oxidation forming **1**. Compound **2** can be produced by the same biosynthetic pathway, with an addition of acetate at C3. The resulting carbonyl group is reduced to alcohol, followed by water elimination and spontaneous tautomerization to originate **2**.

## Discussion

The discovery of bacterially-produced pyrazines from isolates of leaf-cutter ants presents an interesting example of niche-specific chemistry. While ant-derived microbes have been chemically investigated^15,16,26,27^, this study is the first to report convergent chemistry between ants and their associated bacteria. Moreover, biological roles of pyrazines and their effects on ant behavior are well-established. VOCs are commonly used by insects for communication^18,28,29^. Pyrazines, for instance, have been found in ants, phasmids and wasps, playing important roles as alarm, defense, sex and trail pheromones^9,11,30–33^.

Insects can use compounds from their diet as pheromone precursors, as occur with the bark beetles *Ips paraconfusus*, which acquire pheromone precursors from plants^34,35^. Arthropod-associated bacteria can also provide VOCs for insects: the German cockroach *Blattella germanica*^18^ and the locust *Schistocerca gregaria*^19^ use VOCs produced by gut symbiotic bacteria. The pyrazines produced by *S. marcescens* ant-associated bacteria likely contribute to the complex molecular cocktail of pheromones that are used by *A. sexdens rubropilosa* ants. An interesting secondary hypothesis, and one that takes a bacterial perspective, posits that bacteria have evolved to attract their hosts. The trail pheromones are essentially attractants, and free-living forms of the bacteria may produce the pyrazines to initiate a symbiotic relationship with the leaf-cutters. Bacteria in the genus *Serratia* were also identified in colonies of the fungus-growing ants *Atta colombica*, *Apterostigma dentigerum*, *Cyphomyrmex longiscapus* and *Trachymyrmex zeteki*, but their VOCs were not investigated^36,37^. The producer(s) of volatile pyrazines in the *A. sexdens rubropilosa* ants merit subsequent field and laboratory investigations in order to better understand and delineate the insect and possible bacterial contribution to pheromonal communication in leaf-cutter ants.

Despite their important role as trail pheromones, very little is known about the biosynthesis of pyrazines in ants and bacteria. The utilization of L-threonine by *Serratia* results in the transient accumulation of aminoacetone^38^, a metabolite that may represent a direct precursor to **1** and **2**. An aminoacetone oxidase from the human oral microbiome isolate *Streptococcus oligofermentans* has been reported to catalyze the formation of **1** from two equivalents of aminoacetone^39^. Moreover, in a closely related molecule, 3,5-dimethylpyrazin-2-ol (DPO), a threonine dehydrogenase, which catalyzes the formation of 2-amino-3-ketobutyric acid from L-threonine was implicated^14^. It is the spontaneous decarboxylation of 2-amino-3-ketobutyric acid that results in the afore mentioned pool of aminoacetone. An enzyme that utilizes aminoacetone and L-alanine to form the pyrazine DPO, however, is unknown.

Finding that L-threonine induces the ant-isolate bacterium *S. marcescens* 3B2 to produce the trail pheromone pyrazines **1** and **2** is consistent with the high level of incorporation observed in these products. Amino acids often trigger significant physiological responses in bacteria and higher organisms^40–43^. The amino acid L-isoleucine induces epithelial cells to produce β-defensin, which belongs to a family of antimicrobial peptides important to the mammalian innate immune system to combat pathogens^40^. L-proline is an amino acid component of the insect hemolymph, and contributes to the upregulation of the bacterial production of small molecules essential for the virulence of the nematode-associated bacteria^41^. Finally, L-tryptophan produced by a marine algae induce symbiont bacteria to produce the growth promoter auxin indole-3-acetic acid^42,43^. The role and regulation of L-threonine levels in the poison glands of *Atta* are currently unknown, but clearly amino acid availability shapes the distribution of pyrazines produced by the ant-associated *Serratia*.

In summary, *Serratia marcescens* 3B2 isolated from the leaf-cutter ant *Atta sexdens rubropilosa* produces volatile pyrazines, which can vary according the composition of the culturing media. Pyrazines **1** and **2** are known components of its host’s trail pheromone, and pyrazines **4** and **5** are likewise alarm pheromones of other ants^10,11^. The bacterial biosynthesis of **1** and **2** is stimulated by L-threonine, which was found to be the precursor of their 2,5-dimethylpyrazine rings, while the ethyl group of **2** is derived from acetate. The physiological and ecological relevance of these unprecedented findings merits to be further explored in the future.

## Methods

### General method for bacterial isolation and identification

Ant-associated bacteria were isolated by washing ants and plating the aqueous cell suspensions on selective medium^21,22^. We performed two independent isolation experiments, using two *Atta sexdens rubropilosa* nests, collected in the campus of University of São Paulo, Ribeirão Preto, SP, Brazil and maintained in laboratorial conditions (colony 1 and colony 2), according to SISBIO authorization 46555-5, and CNPq process 010936/2014-9. For the first isolation process, three *Atta sexdens rubropilosa* ants from colony 1 were transferred to a sterile 1.5 mL Eppendorf tube containing 750 µL of 0.9% NaCl isotonic aqueous solution. The tube was vortexed during approximately 1 min and 100 µL of washing solution were inoculated on ISP-2 agar plates (80 mm; 4% of yeast extract, 10% of malt extract, 4% of glucose and 20% of agar in distilled water) added with antifungal agents (nystatin 0.04 g.L^−1^ and cycloheximide 0.05 g.L^−1^). The plates were incubated at 30 °C and the grown bacterial colonies were isolated on ISP-2 agar plates.

For the second isolation procedure, ants from colony 2 were surveyed for bacterial strains. Three ants were surface sterilized using a modified version of a previously methodology used by our group^23^. Each ant was submerged in 70% ethanol during two minutes, followed by 5% of sodium hypochlorite for one minute, 70% of ethanol during one minute and rinsed three times with sterile water. The ants were crushed and transferred to ISP-2 agar plates added with antifungal agents. A non-surface sterilized ant was transferred into a sterile 1.5 mL Eppendorf tube containing 750 µL of 0.9% NaCl isotonic aqueous solution, the tube was vortexed during approximately 1 min and 100 µL of washing solution were inoculated on ISP-2 agar plate with antifungal agents. Additionally, three ants were also surface sterilized and the poison glands were dissected in aseptic conditions. The poison glands were inoculated on ISP-2 agar plates to isolate bacterial colonies. The bacterial isolation was performed according the procedure done for colony 1. Strains 3B2 and 3B4 were isolated in the first isolation procedure, while in the second experiment, strains ASLIM1, ASLIM5, ASLIM6, ASLIM7 were isolated from sterile ants, strain ASLFLB2 from a non-sterile ant, and strain AS1GV1B1 from a poison gland. Stocks of the eight bacterial isolates were prepared in ISP-2 or M9 liquid media with 20% of glycerol and maintained in −80 °C freezer.

The bacterial identification was carried out by sequencing the 16S rRNA, using the primers 27F and 1492R^44^, and the sequences were compared with GenBank sequences by BLASTn analysis. The *S. marcescens* 3B2 16S rRNA sequence has been deposited in the GenBank, and can be accessed using the GenBank accession number MF450442.

### General procedure for bacterial cultivation

Aliquots of glycerol stocks (100 µL) were inoculated on ISP-2 agar plates (80 mm) containing approximately 25 mL of medium. Empty Petri plates were sealed on top of the inoculated plates, and incubated for three days at 30 °C. The polydimethylsiloxane GC-MS fibber was exposed inside of these plates during approximately 60 min to adsorb the volatile organic compounds (VOCs), which were analyzed by GC-MS.

*Serratia marcescens* 3B2 was also cultivated using the synthetic medium M9 added with 0.2% of glucose and 1.5% of the amino acids L-threonine, L-serine, L-valine and L-alanine in independent experiments. The amino acid L-threonine was also added in the M9 medium at 0.5%, 1.0%, 1.5% and 2.0%. All amino acids were dissolved with distilled water and filter sterilized before addition to the culture medium.

M9 medium (100 mL): 20 mL of M9 5X, 2 mL of glucose 20%, 200 µL of MgSO_4_ 1 M, 10 µL of CaCl_2_ 1 M, the final volume was adjusted with 100 mL of distilled water and the solution was filter sterilized. To parepare M9 agar medium, 20 g of agar in 40 mL of distilled water were autoclaved and mixed with filter sterilized: 20 mL of M9 5X, 2 mL of glucose 20%, 200 µL of MgSO_4_ 1 M, 10 µL of CaCl_2_ 1 M, and the final volume was adjusted with 100 mL of sterile distilled water.

### Chemical analysis

The GC-MS analyses were performed on a GC-2010 Plus Shimadzu coupled with DB-5 MS column (30 m × 0.25 mm × 0.25 µm). The polydimethylsiloxane fibber with the VOCs was exposed during three minutes into the GC-MS injector at 250 °C. The oven temperature started with 50 °C during 5 min, followed by an increase of 3 °C/min to 180 °C and held for 10 min. Analyses were carried out in splitless injection mode, with helium as carrier gas, ion source temperature of 250 °C and 70 eV for sample ionization. VOCs were identified by comparing the GC-MS spectra with those of databases Wiley 7 and NIST 08, analyzing fragmentation patterns and comparing retention times and mass spectra with Sigma-Aldrich standards of pyrazines **1** and **2**.

The LC-MS analyses were performed on an Accela UHPLC (Thermo Scientific, Carlsbad, CA, USA) interfaced with an ExactiveTM Plus Orbitrap mass spectrometer (MS) (Thermo Scientific). The Kinetex C_18_ (150 × 2.10 mm, 1.7 µm) column was eluted with mixtures of acetonitrile (CH_3_CN) and water, both add with 0.1% of formic acid, with the following gradient at flow rate of 400 µL/min: 0–15 min of gradient from 5% of CH_3_CN to 50% of CH_3_CN, 15–20 min of isocratic 50% of CH_3_CN, 20–30 min of gradient from 50% of CH_3_CN to 100% of CH_3_CN, 30–35 min of isocratic 100% of CH_3_CN and 35–40 min of gradient from 100% of CH_3_CN to 5% of CH_3_CN. Samples were analyzed in positive ESI mode over the mass range 100 to 1500 Da, mass accuracy was bellow 3 ppm, capillary temperature was 320 °C and spray voltage was 3.6 kV.

### Isotope feeding experiments

The bacterial culturing for isotope feeding experiments were performed using 10 mL GC-MS vial added with 2 mL of culture media. The medium M9 agar supplemented with 0.2% of glucose, 1.5% of L-threonine and 0.5% of L-alanine was mixed with labelled or non-labelled precursors dissolved in MilliQ water. To prepare the culture media, 0.04 g of agar were mixed with 700 µL of MilliQ water into 10 mL SPME vials and they were sterile autoclaved. After that, the precursors were dissolved with 900 µL of sterile water and they were mixed with 400 µL of M9 5X salts, 40 µL of 20% aqueous glucose solution, 4 µL of MgSO_4_ 1 M and 0.2 µL of CaCl_2_ 1 M, which were finally filter sterilized and mixed with the sterile agar. Aliquots (100 µL) of *Serratia marcescens* 3B2 glycerol stocks on M9 were inoculated into each 10 mL GC-MS vials containing the culture media, which were sealed and incubated for three days at 30 °C. The VOCs were extracted using a polydimethylsiloxane GC-MS fibber exposed inside of these vials during approximately 60 min, and the VOCs were analyzed by GC-MS. Isotope-labelled precursors L-[U-^13^C,^15^N]-threonine, U-^13^C-sodium acetate, 3-^13^C-L-alanine and ^15^N-L-alanine were purchased from Sigma-Aldrich.

### Purification of compound 6

*S. marcescens* 3B2 was cultivated onto 20 plates of ISP-2 agar (100 mL per 150 × 15 mm Petri dish) for 14 days at 30 °C and the culture medium was extracted with dichloromethane. The resulting extract was fractionated using solid-phase extraction (silica 2 g, Fisher cartridge) eluted with the following solvent gradient: 100% of hexane, 80% of hexane and 20% of ethyl acetate, 60% of hexane and 40% of ethyl acetate, 40% of hexane and 60% of ethyl acetate, 20% of hexane and 80% of ethyl acetate and 100% of ethyl acetate. Compound **6** (3.8 mg) was isolated from the fraction with 80% of hexane and 20% of ethyl acetate, which was purified using semi-preparative HPLC (Agilent 1200 series HPLC-DAD) coupled to a normal phase column (Phenomenex Luna 5 µm silica, 250 × 10 mm) and eluted with the isocratic mobile phase of 98% hexane and 2% of isopropanol (3 mL/min).

## Electronic supplementary material

Supplementary Information
