# Supplementary material for: Pyrazines from bacteria and ants: convergent chemistry within an ecological niche
Source: Sci Rep. 2018 Feb 7;8:2595. doi: 10.1038/s41598-018-20953-6 (PMC5803209; doi:10.1038/s41598-018-20953-6)
Supplement: Supplementary file 1 — Supplementary Information [file 41598_2018_20953_MOESM1_ESM.pdf]

Electronic Supporting Information for:

Pyrazines from bacteria and ants: convergent chemistry within an ecological niche

Eduardo A. Silva-Junior,[a] Antonio C. Ruzzini[b#], Camila R. Paludo[a], Fabio S. Nascimento[c], Cameron R. Currie[d], Jon Clardy[b], and Mônica T. Pupo*[a]

[a]Universidade de São Paulo, Faculdade de Ciências Farmacêuticas de Ribeirão Preto, Ribeirão Preto, 14040903, Brazil.
[b]Harvard Medical School, Department of Biological Chemistry and Molecular Pharmacology, Boston, MA 02115, United States of America.
[c]Universidade de São Paulo, Departamento de Biologia, Faculdade de Filosofia, Ciências e Letras de Ribeirão Preto, Ribeirão Preto, 14040901, Brazil.
[d]University of Wisconsin, Department of Bacteriology, Madison, WI 53706, United States of America.
[#]current address: Department of Veterinary Microbiology, University of Saskatchewan, Saskatchewan, SK, Canada, S7N 5B4.

* Correspondence should be addressed to Mônica T. Pupo (mtpupo@fcfrp.usp.br)

Figure S1. GC-MS analysis of VOCs produced by strains 3B2 in A, 3B4 in B, ASLFLB2 in C, ASLIM1 in D, ASLIM5 in E, ASLIM6 in F, ASLIM7 in G and AS1GV1B1 in H.

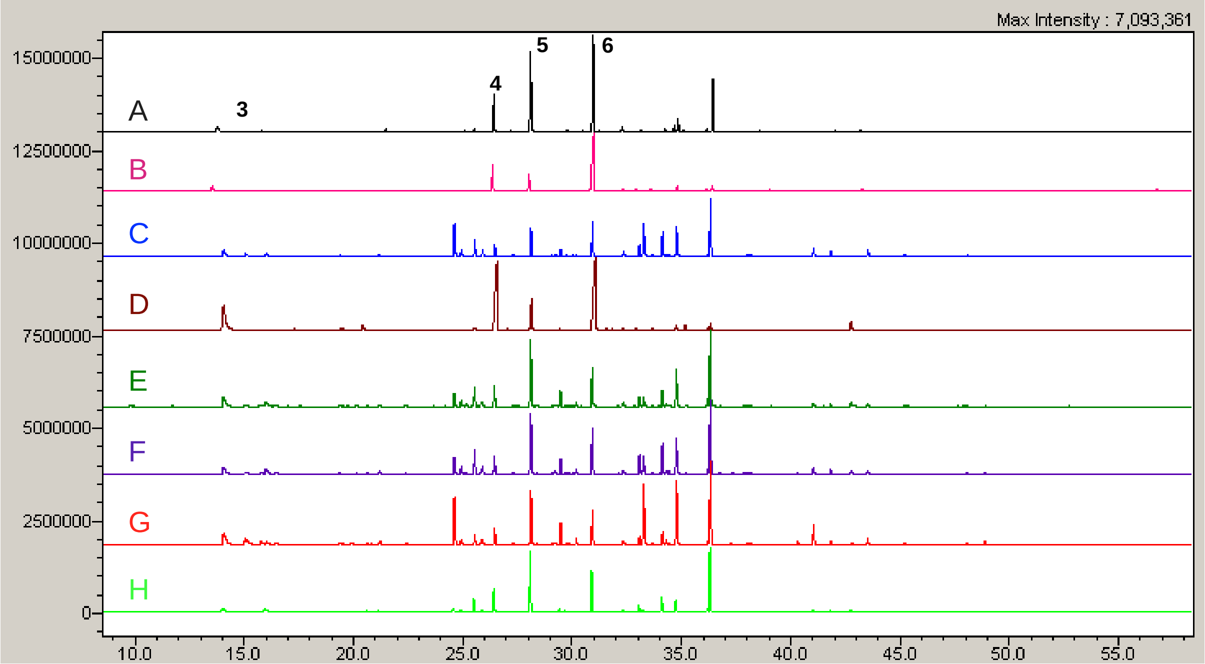

Figure S2. GC-MS spectra of compound **3** in A, compound **4** in B, compound **5** in C and compound **6** in D produced by *Serratia marcescens* 3B2 cultivated on ISP-2 agar medium during three days at 30 ^{o}C.

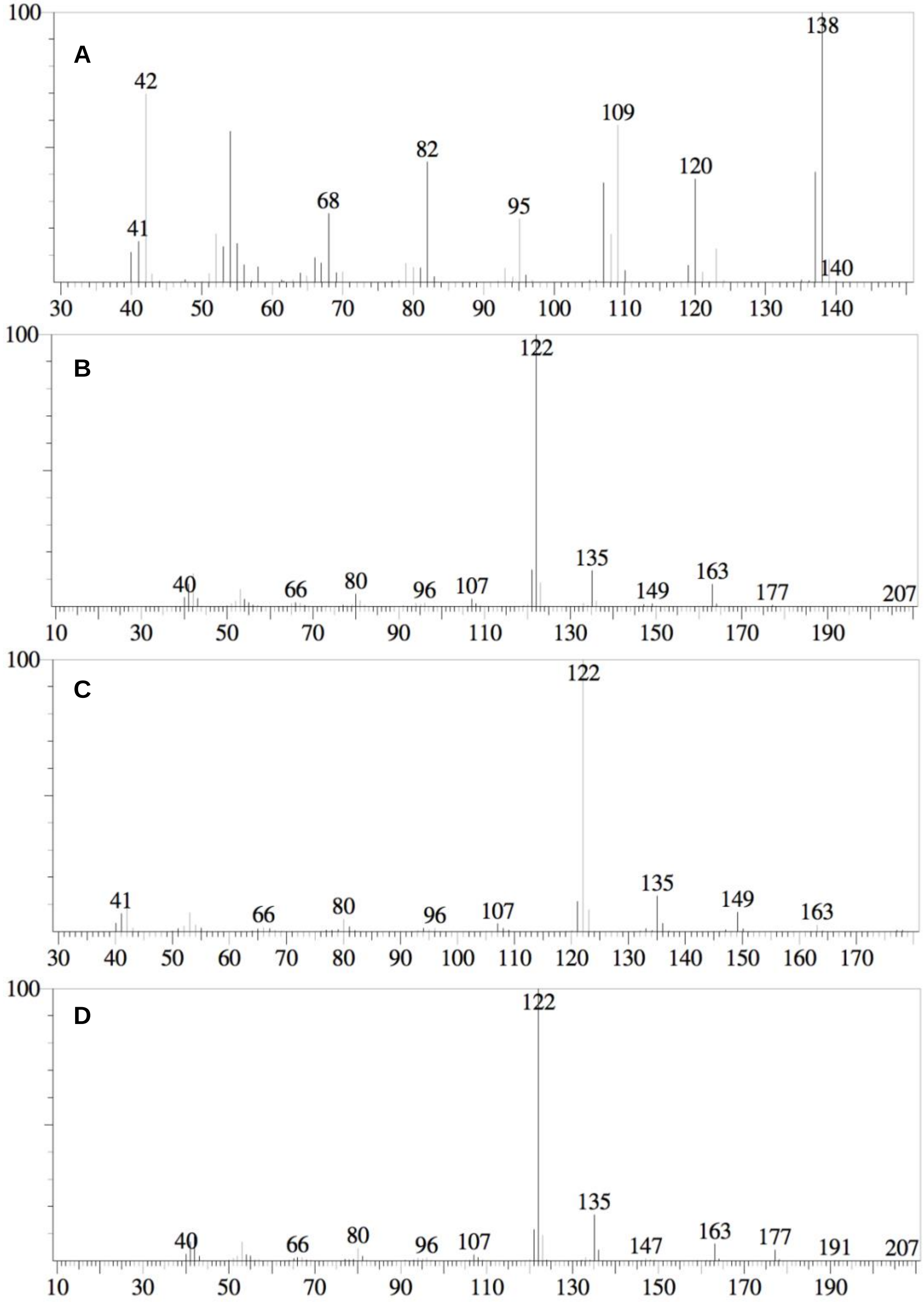

Figure S3. Proposed GC-MS fragmentation pathways for the alkyl chains of compounds **4**, and **5** and **6** (A) and formation of the base ion with *m/z* 122 (B).

A)

Compound **4** (*m/z* 178)

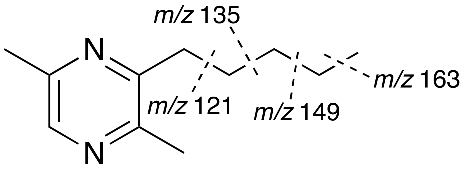

Compound **5** (*m/z* 178)

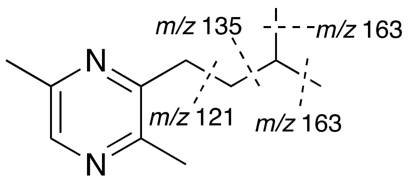

Compound **6** (*m/z* 192)

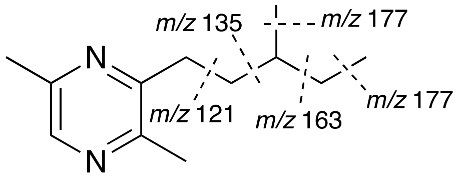

B)

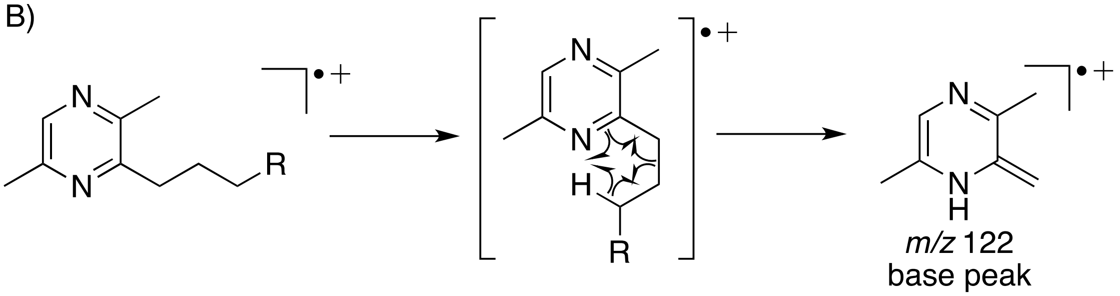

Figure S4. Proposed GC-MS fragmentation pathways for pyrazine **2**.

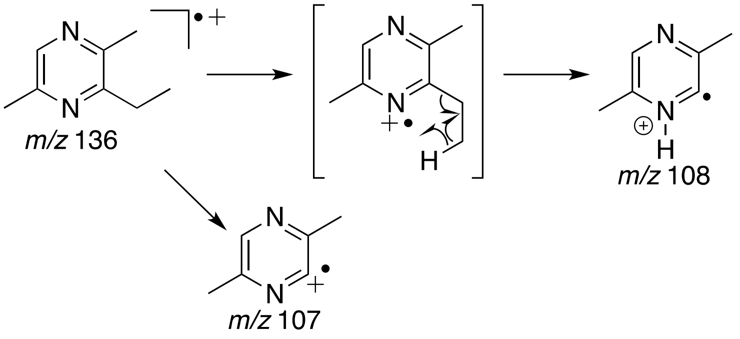

Figure S5. GC-MS spectra of compound **2** produced by *Serratia marcescens* 3B2 cultivated on M9 agar medium supplemented with 0.2% of glucose and 1.5% of L-threonine.

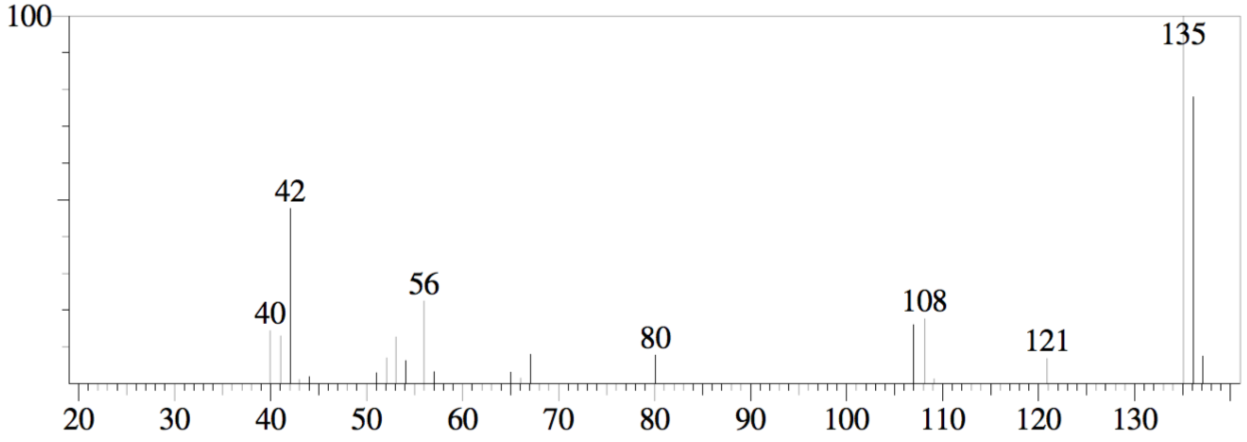

Table S1. NMR spectral data for compound **6** ($CDCl_3$, 500 MHz).

**Position**	**δ ^{13}C***	**^{1}H δ (m; *J* em Hz)**	**H→C *g*HMBC**
2	148.4	-	-
3	155.4	-	-
5	150.2	-	-
6	140.0	8.15 (1H; *s*)	150.2; 148.4; 155.4
2-Me	20.7	2.52 (3H, *s*)	148.4; 155.4
5-Me	20.6	2.48 (3H; *s*)	150.2; 140.0
1’	32.5	2.79 (1H; *m*)	155.4; 148.4; 35.4
		2.72 (1H; *m*)	155.4; 148.4; 35.4
2’	35.4	1.44 (2H; *m*)	35.0
3’	35.0	1.66 (1H; *m*)	18.6; 28.9; 155.4
3’-Me	18.6	0.95 (3H; *d*; J=6.1)	28.9; 35.4; 35.0
4’	28.9	1.39 (1H; *m*)	35.0
		1.25 (1H; *m*)	35.0; 18.6
5’	10.9	0.89 (3H; *t*; J=7.5)	28.9; 35.0

* ^{13}C values were extracted from *g*HMBC and *g*HSQC spectra.

Figure S6. ^{1}H NMR spectrum of compound **6** (600 MHz, $CDCl_3$).

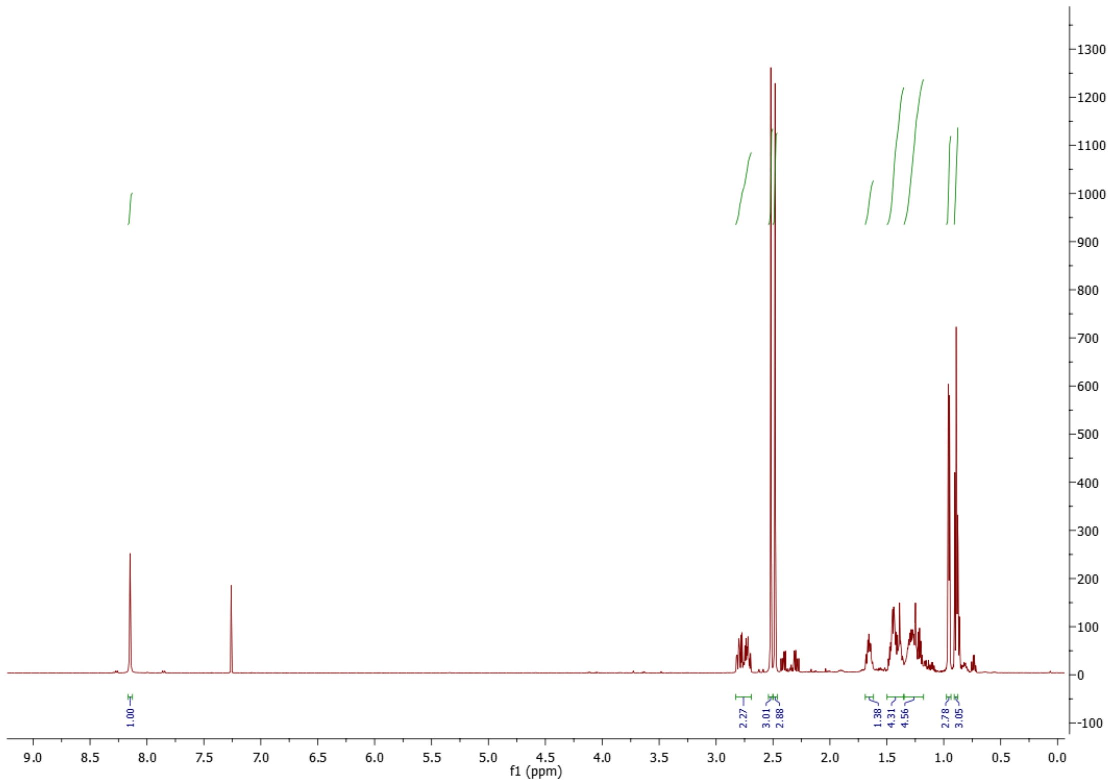

Figure S7. *g*HSQC spectrum of compound **6** (600 MHz, $CDCl_3$).

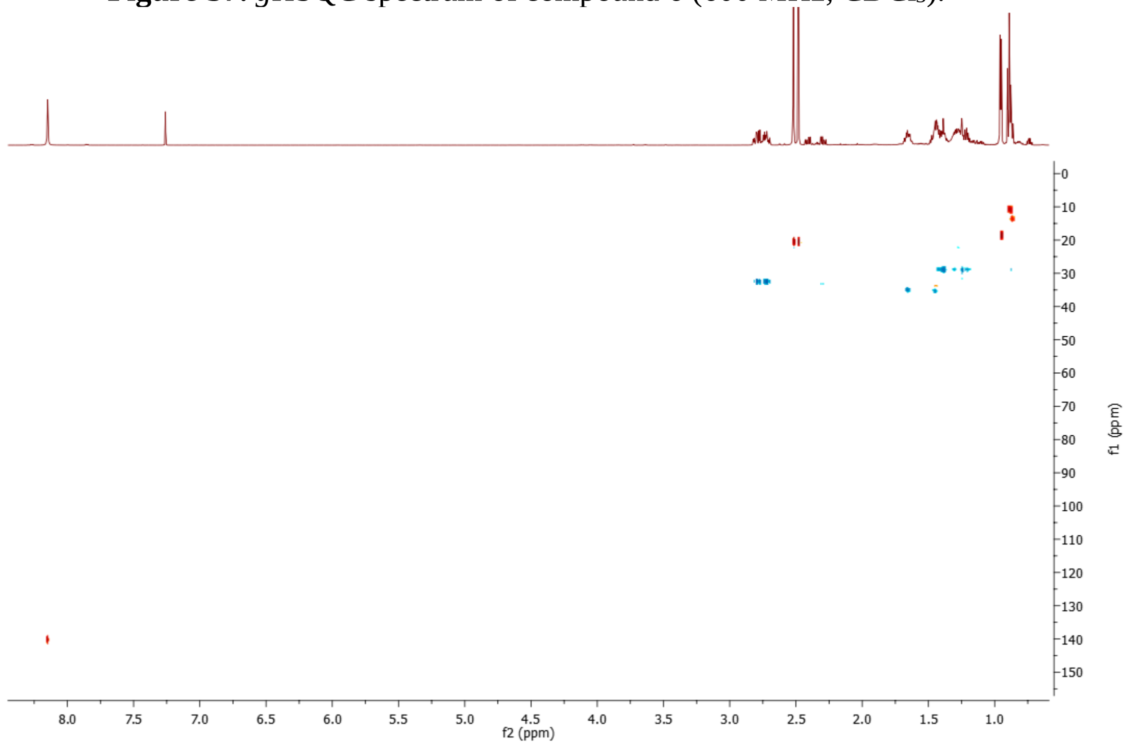

Figure S8. *g*HMBC spectrum of compound **6** (600 MHz, $CDCl_3$).

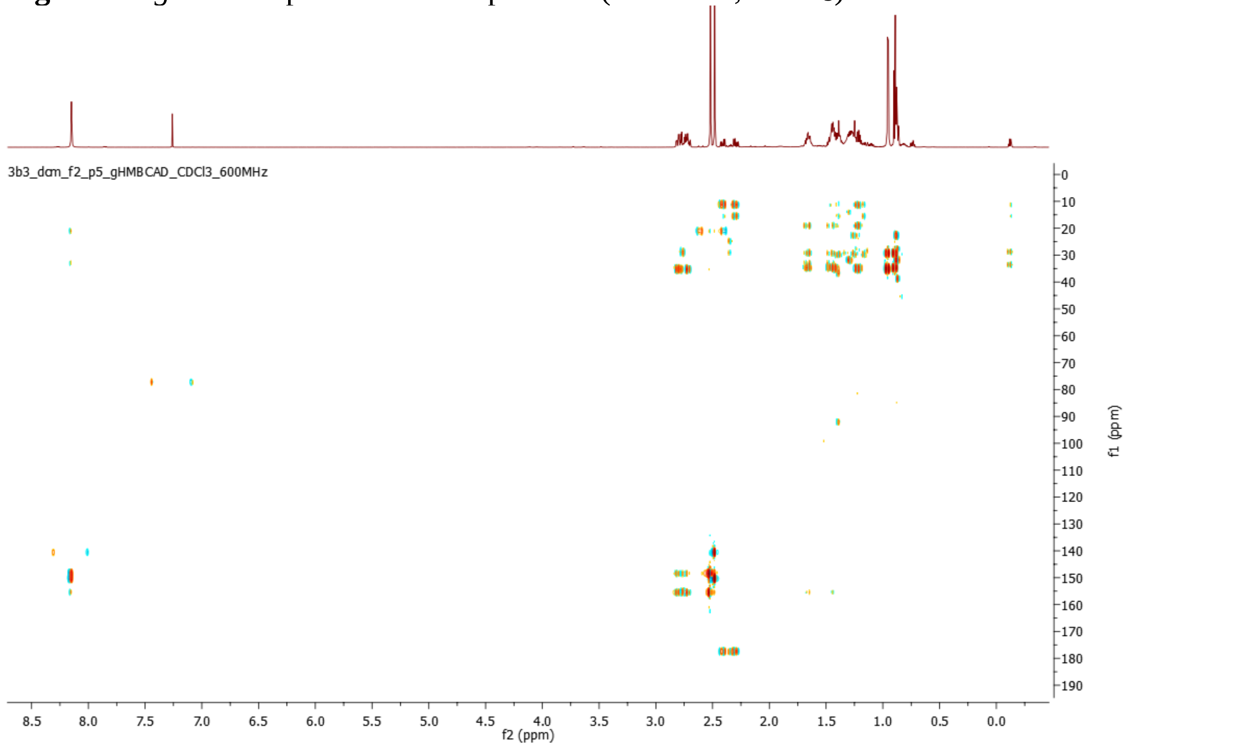

Figure S9. gCOSY spectrum of compound **6** (600 MHz, $CDCl_3$).

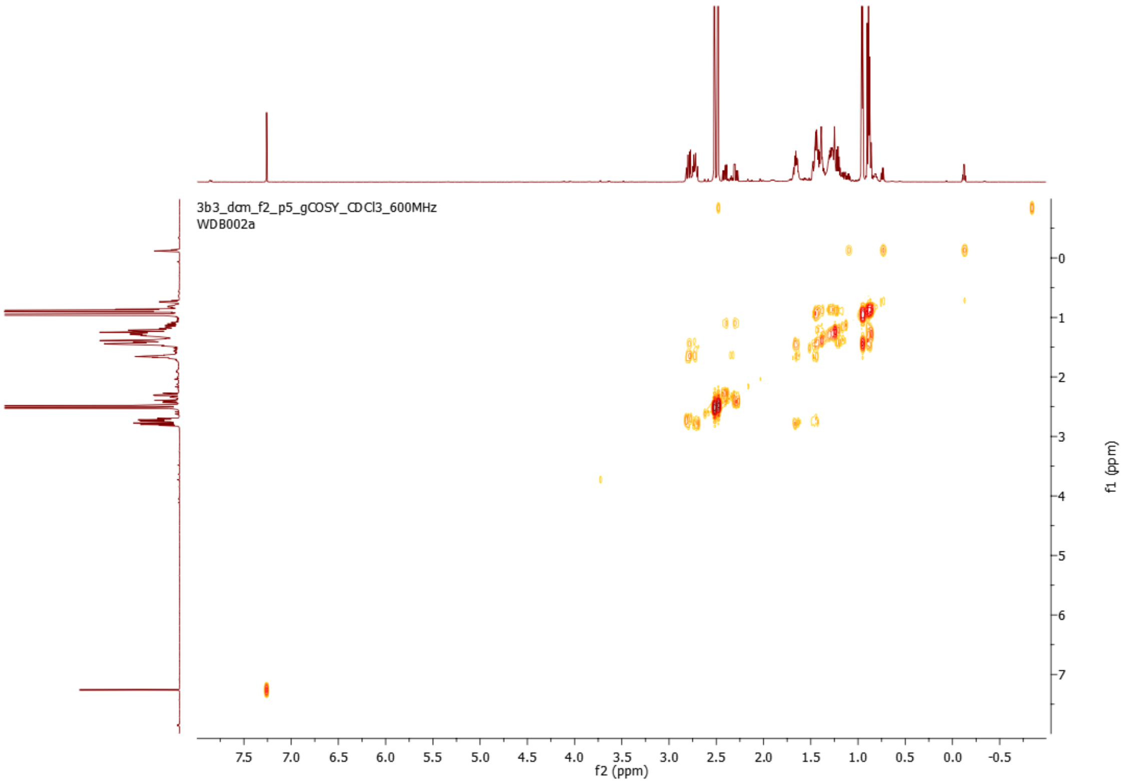

Figure S10. HR-ESI-MS spectrum of compound **6** at positive ion monitoring mode. $[M+H]^+$ $C_{12}H_{21}N_2$ (*m/z* 193.1694), error: 2.6 ppm.

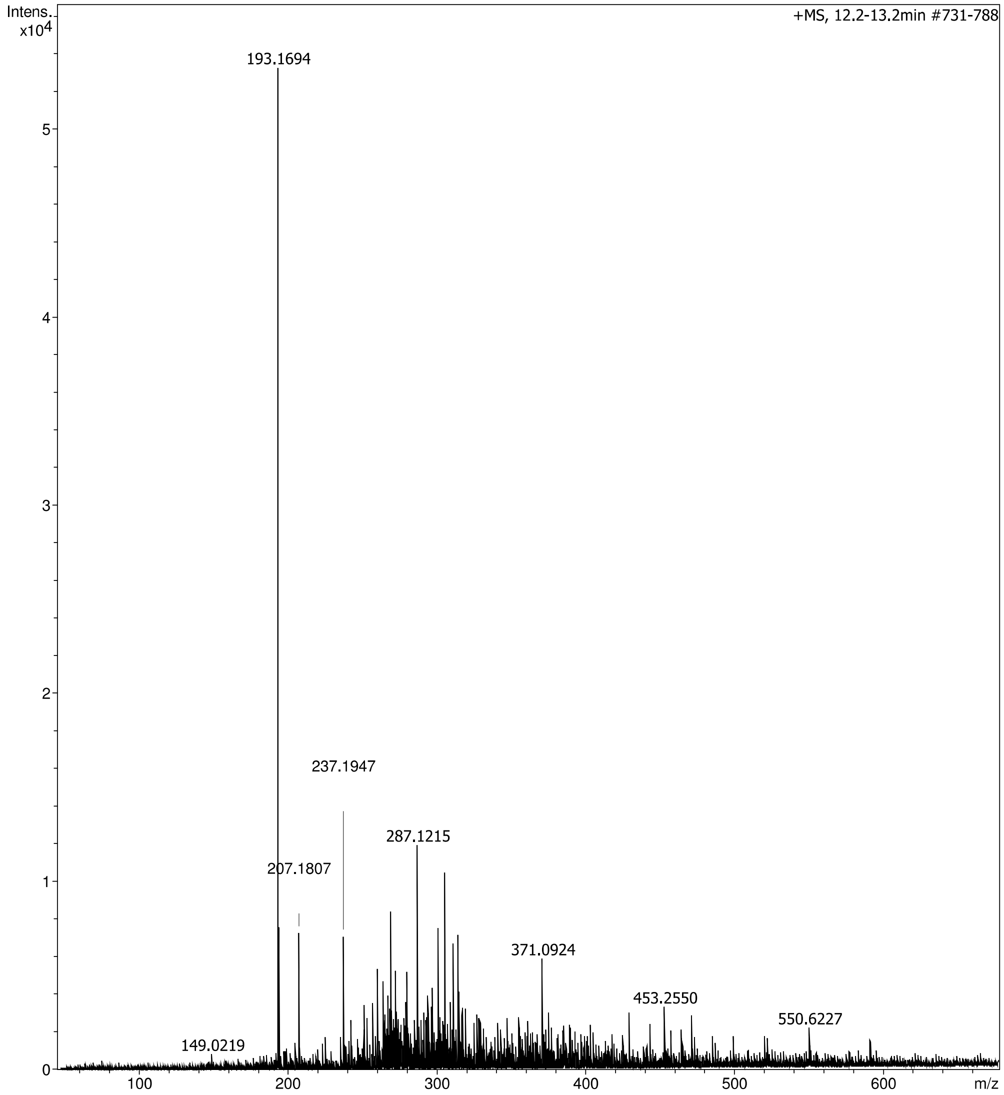

Figure S11. GC-MS analysis of VOCs from five dissected poison glands in A, five gasters of *Atta sexdens rubropilosa* in B, Sigma-Aldrich standards of **1** in C and 3-ethyl-2,(5 or 6)-dimethylpyrazine mixture of isomers in D. 2,5-dimethylpyrazine (**1**) peaks are highlighted with blue rectangle and 3-ethyl-2,5-dimethylpyrazine (**2**) with red rectangle.

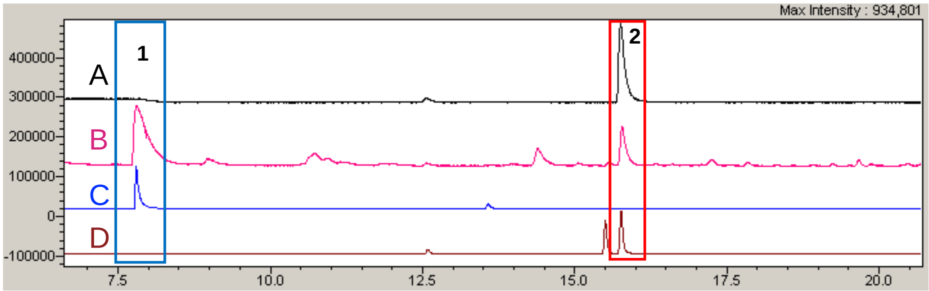

Figure S12. GC-MS spectra of 2,5-dimethylpyrazine (**1**), molecular ion with *m/z* 108, from five dissected poison glands in A, five gasters of *Atta sexdens rubropilosa* in B, Sigma-Aldrich standard of **1** in C.

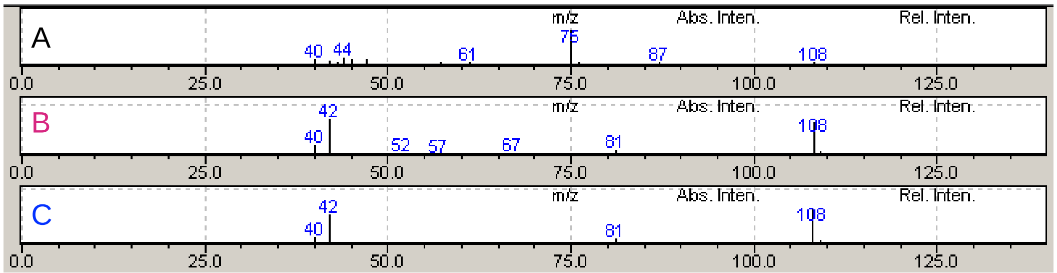

Figure S13. GC-MS spectra of 3-ethyl-2,5-dimethylpyrazine (**2**), molecular ion with *m/z* 136, from five dissected poison glands in A, five gasters of *Atta sexdens rubropilosa* in B, and Sigma-Aldrich standard of **1** in C.

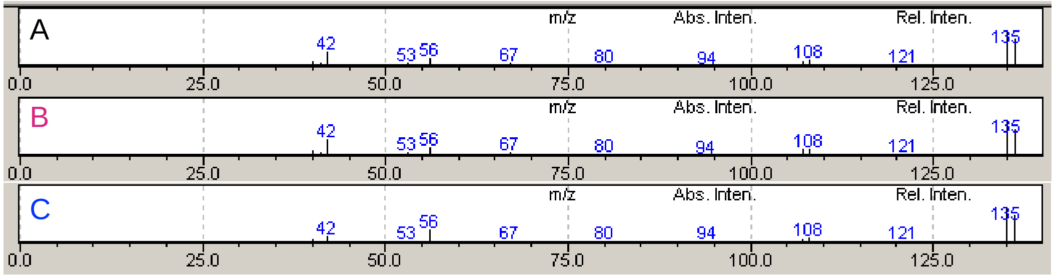

Figure S14. GC-MS analysis of VOCs produced by *Serratia marcescens* 3B2 cultivated on M9 agar medium supplemented with 0.2% of glucose and in A, and cultivated on the same medium added with 1.5% of L-threonine in B, 2.0% of L-threonine in C, 1.5% of L-valine in D, 1.5% of L-serine in E and 1.5% of L-alanine in F. 2,5-dimethylpyrazine (**1**) peaks are highlighted with blue rectangle and 3-ethyl-2,5-dimethylpyrazine with red rectangle (**2**).

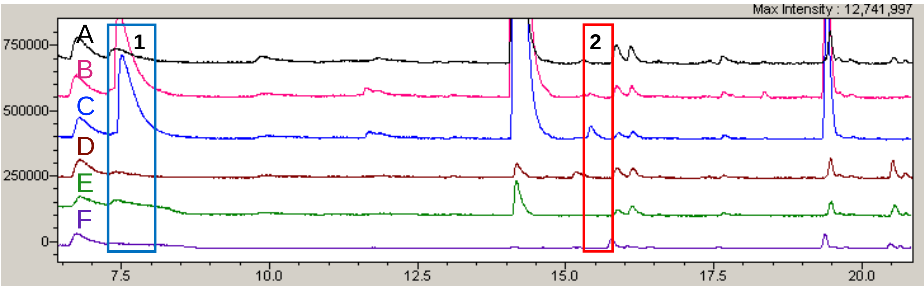

Figure S15. GC-MS spectra of 2,5-dimethylpyrazine (**1**), retention time 7.5 min and molecular ion with *m/z* 108, produced by *S. marcescens* 3B2 cultivated on M9 agar medium supplemented with 0.2% of glucose and 1.5% of L-threonine in B and same medium with 2.0% of L-threonine in C. Compound **1** was not detected on cultures of *S. marcescens* 3B2 on M9 agar medium supplemented with 0.2% of glucose in A and M9 agar medium supplemented with 0.2% of glucose and 1.5% of L-valine in D, 1.5% of L-serine in E and 1.5% of L-alanine in F.

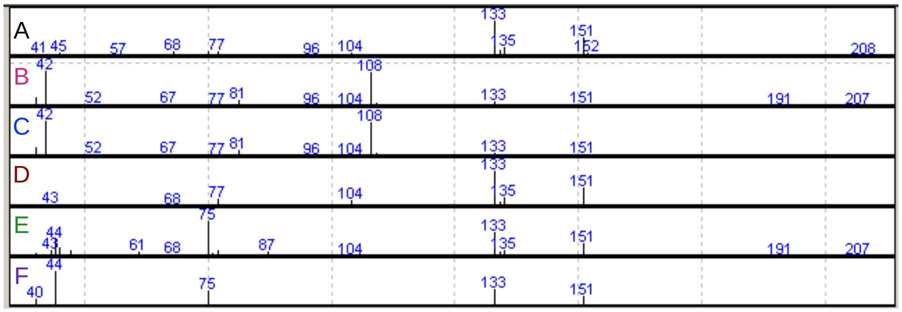

Figure S16. GC-MS spectra of 3-ethyl-2,5-dimethylpyrazine (**2**), retention time 15.4 min and molecular ion with *m/z* 136, produced by *S. marcescens* 3B2 cultivated on M9 agar medium supplemented with 0.2% of glucose and 1.5% of L-threonine in B and same medium with 2.0% of L-threonine in C. Compound **2** was not detected on cultures of *S. marcescens* 3B2 on M9 agar medium supplemented with 0.2% of glucose in A and M9 agar medium supplemented with 0.2% of glucose and 1.5% of L-valine in D, 1.5% of L-serine in E and 1.5% of L-alanine in F.

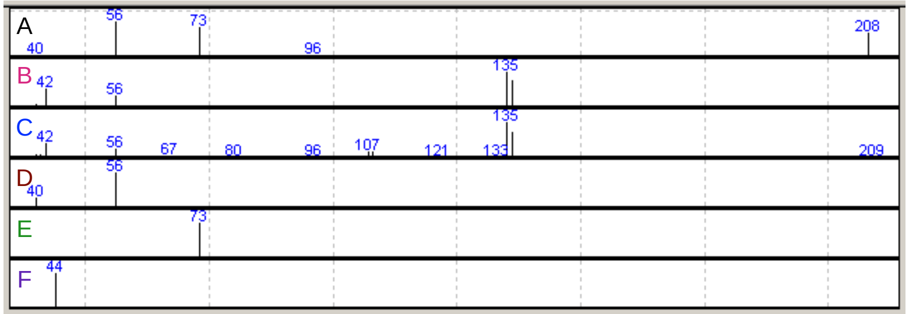

Figure S17. GC-MS analysis of VOCs produced by *Serratia marcescens* 3B2 cultivated on M9 agar medium supplemented with 0.2% of glucose in A, M9 agar medium supplemented with 0.2% of glucose and 0.5% of L-threonine in B, 1.0% of L-threonine in C, 1.5% of L-threonine in D and 2.0% of L-threonine in E.

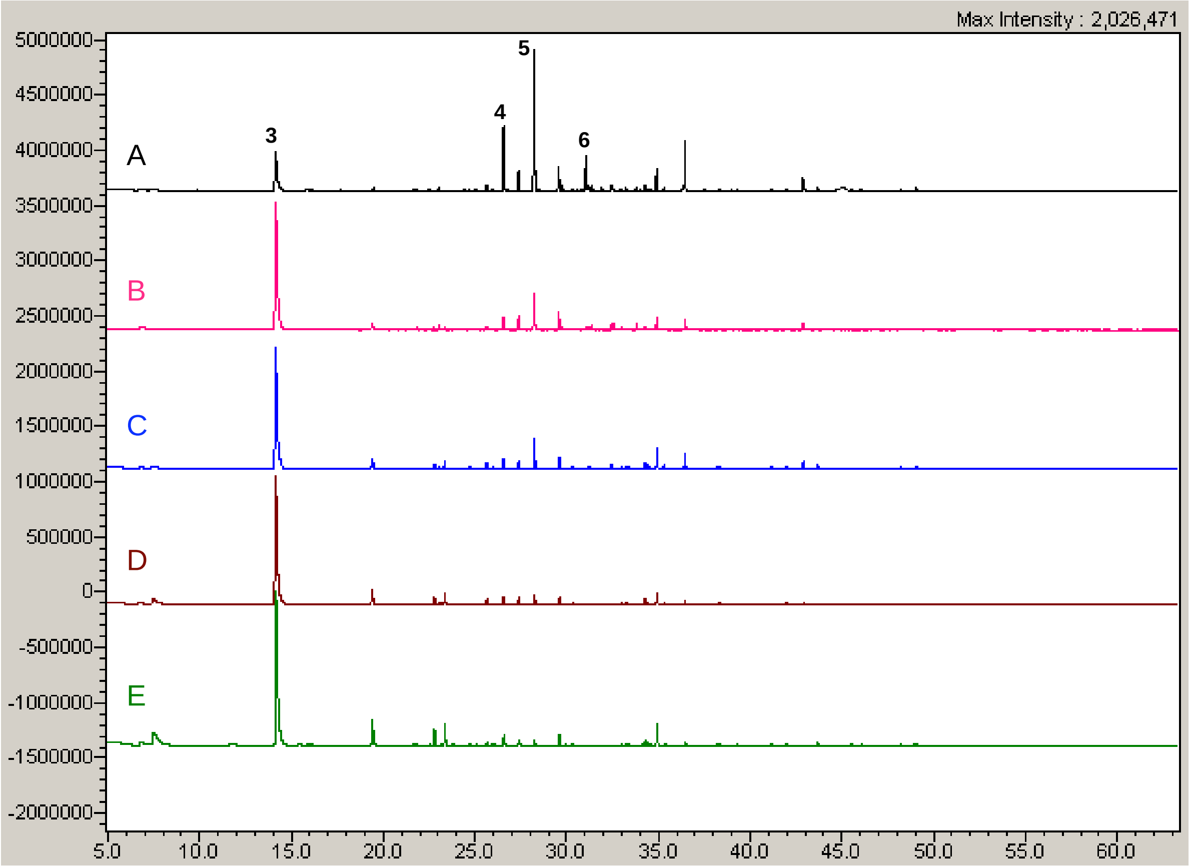

Figure S18. GC-MS analysis of VOCs produced by *Serratia marcescens* 3B2 cultivated on M9 agar medium supplemented with 0.2% of glucose in A, and cultivated on the same medium added with 0.5% of L-threonine in B, 1.0% of L-threonine in C, 1.5% of L-threonine in D and 2.0% of L-threonine in E. 2,5-dimethylpyrazine (**1**) peaks are highlighted with blue rectangle and 3-ethyl-2,5-dimethylpyrazine (**2**) with red rectangle.

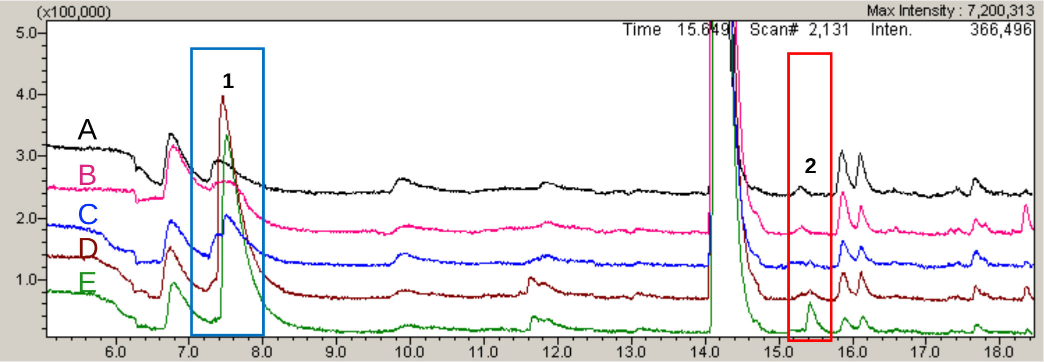

Figure S19. GC-MS spectra of 2,5-dimethylpyrazine (**1**), retention time 7.5 min and molecular ion with *m/z* 108, produced by *S. marcescens* 3B2 cultivated on M9 agar medium supplemented with 0.2% of glucose and 1.0% of L-threonine in C, 1.5% of L-threonine in D and 2.0% of L-threonine in E. Compound **1** was not detected when the bacterium was cultivated on M9 agar medium supplemented with 0.2% of glucose in A and M9 agar medium supplemented with 0.2% of glucose and 0.5% of L-threonine in B.

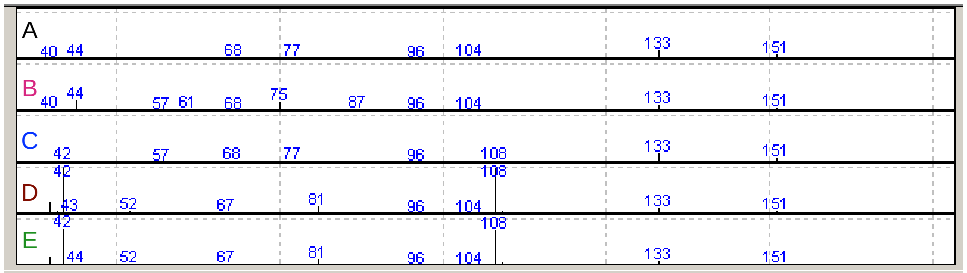

Figure S20. GC-MS spectra of 3-ethyl-2,5-dimethylpyrazine (**2**), retention time 15.4 min and molecular ion with *m/z* 136, produced by *Serratia marcescens* 3B2 cultivated on M9 agar medium supplemented with 0.2% of glucose and 1.0% of L-threonine in C, 1.5% of L-threonine in D and 2.0% of L-threonine in E. Compound **2** was not detected when the bacterium was cultivated on M9 agar medium supplemented with 0.2% of glucose in A and M9 agar medium supplemented with 0.2% of glucose and 0.5% of L-threonine in B.

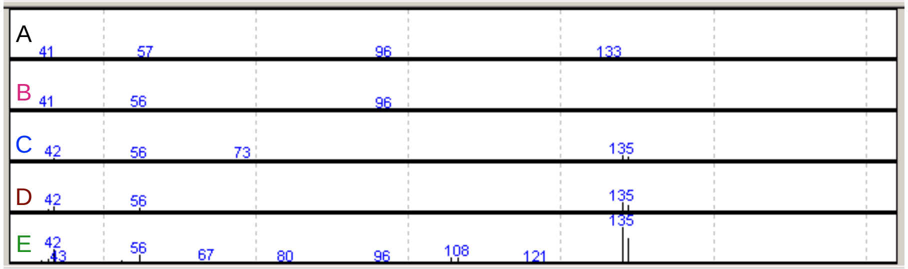

Figure S21. GC-MS analysis of VOCs produced by *Serratia marcescens* 3B2 cultivated on M9 agar medium supplemented with 0.2% of glucose, 1.5% of L-threonine and 0.5% of L-alanine in A and the control medium in B. 2,5-dimethylpyrazine (**1**) peak is highlighted with blue rectangle and 3-ethyl-2,5-dimethylpyrazine (**2**) with red rectangle.

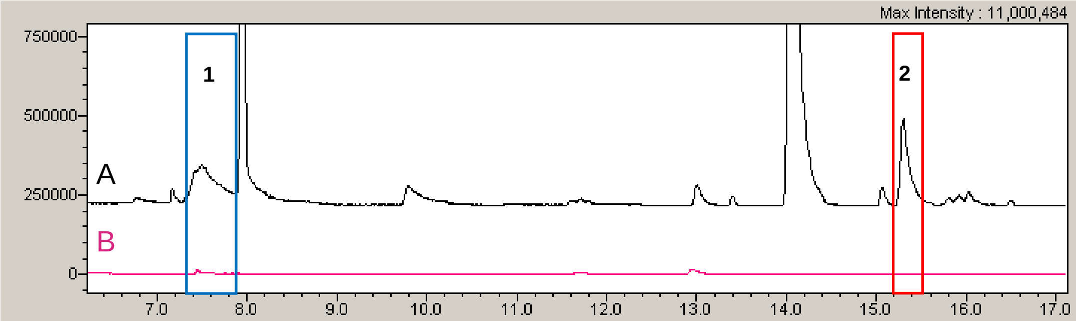

Figure S22. GC-MS spectra of 2,5-dimethylpyrazine (**1**), retention time 7.5 min and molecular ion with *m/z* 108, produced by *Serratia marcescens* 3B2 cultivated on M9 agar medium supplemented with 0.2% of glucose, 1.5% of L-threonine and 0.5% of L-alanine, in A. GC-MS spectra of VOCs from the culture media in 7.5 min showing the absence of **1**, in B.

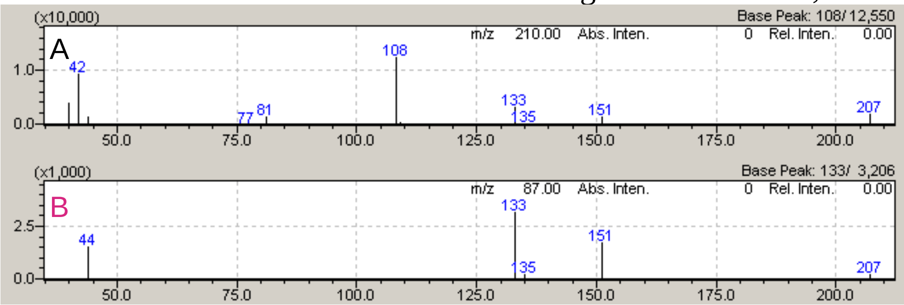

Figure S23. GC-MS spectra of 3-ethyl-2,5-dimethylpyrazine (**2**), retention time 15.4 min and molecular ion with *m/z* 136, produced by *Serratia marcescens* 3B2 cultivated on M9 agar medium supplemented with 0.2% of glucose, 1.5% of L-threonine and 0.5% of L-alanine in A. GC-MS spectra of VOCs from the culture media in 15.4 min showing the absence of **2**, in B.

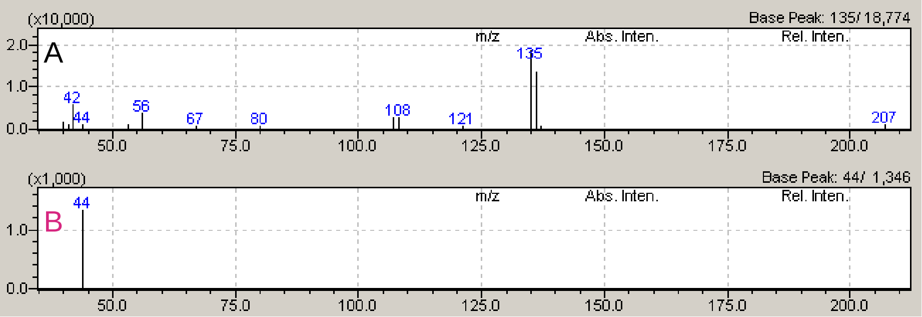

Figure S24. Extracted ion chromatograms from UHPLC-ESI-HRMS analyses of 2,5-dimethylpyrazine (**1**) in A and 3-ethyl-2,5-dimethylpyrazine (**2**) in B produced by *Serratia marcescens* 3B2 cultivated on M9 agar medium supplemented with 0.2% of glucose, 1.5% of L-threonine and 0.5% of L-alanine.

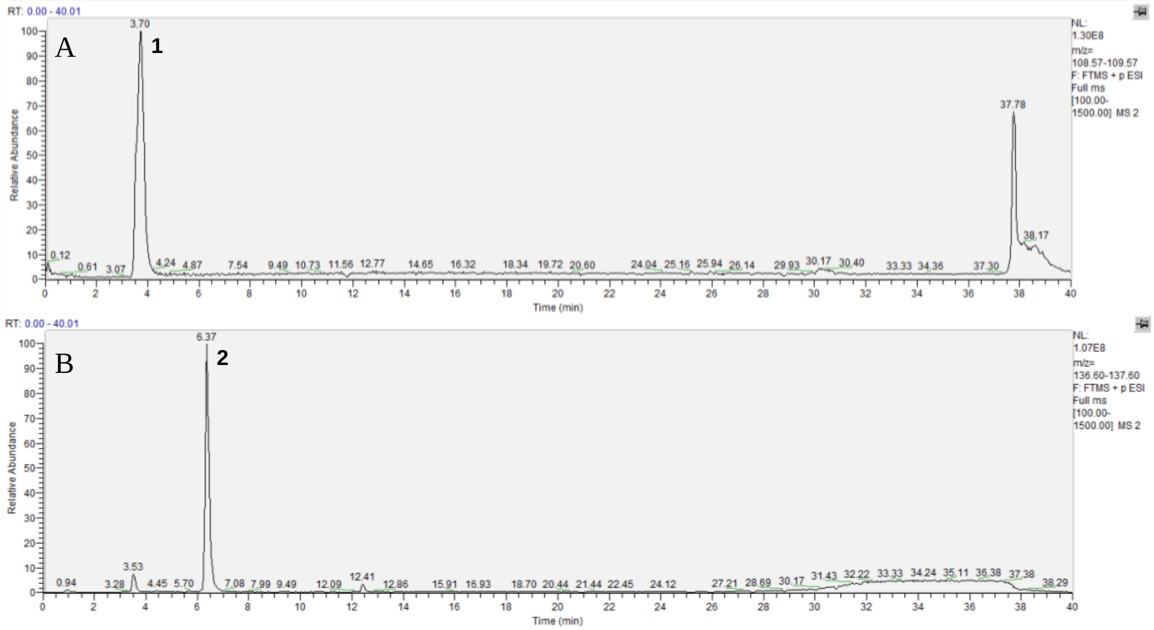

Figure S25. High resolution mass spectra of 2,5-dimethylpyrazine (**1**) in A and 3-ethyl-2,5-dimethylpyrazine (**2**) in B produced by *Serratia marcescens* 3B2 cultivated on M9 agar medium supplemented with 0.2% of glucose, 1.5% of L-threonine and 0.5% of L-alanine. $[M+H]^+$ $C_6H_9N_2$ with *m/z* 109.076 and $[M+H]^+$ $C_8H_{13}N_2$ with *m/z* 137.107.

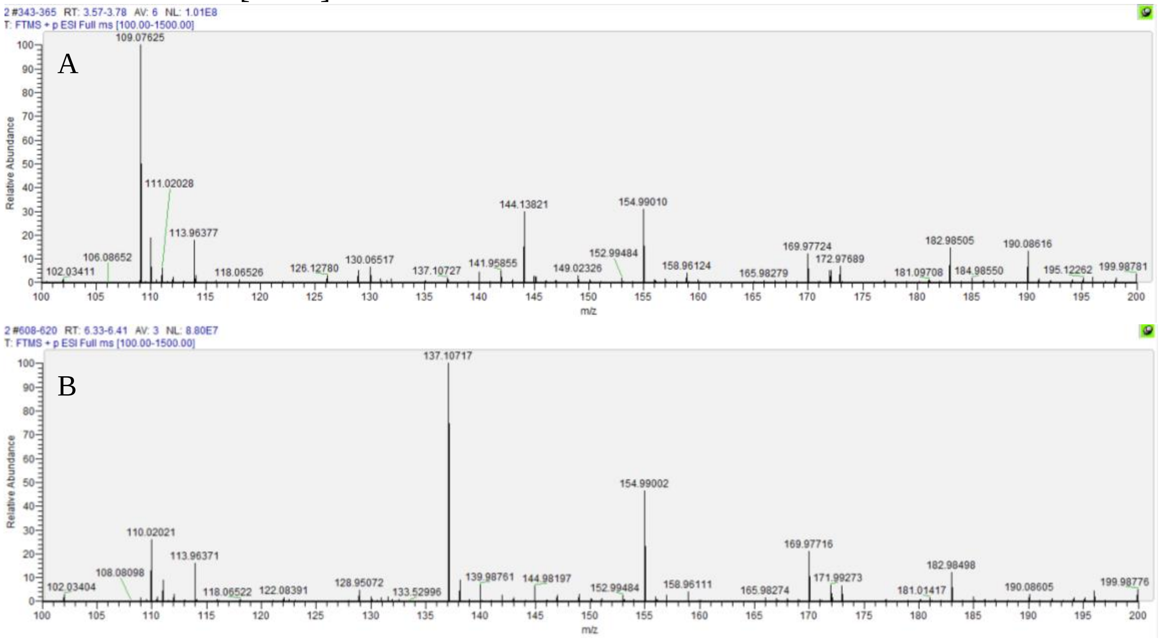

Figure S26. GC-MS analysis of VOCs produced by *Serratia marcescens* 3B2 cultivated on M9 agar medium supplemented with 0.2% of glucose, 0.5% of L-alanine and 1.5% of non-labelled L-threonine in A and cultivated on the same medium added with L-[U-^{13}C,^{15}N]-threonine in B. 2,5-dimethylpyrazine (**1**) peaks are highlighted with blue rectangle and 3-ethyl-2,5-dimethylpyrazine (**2**) with red rectangle.

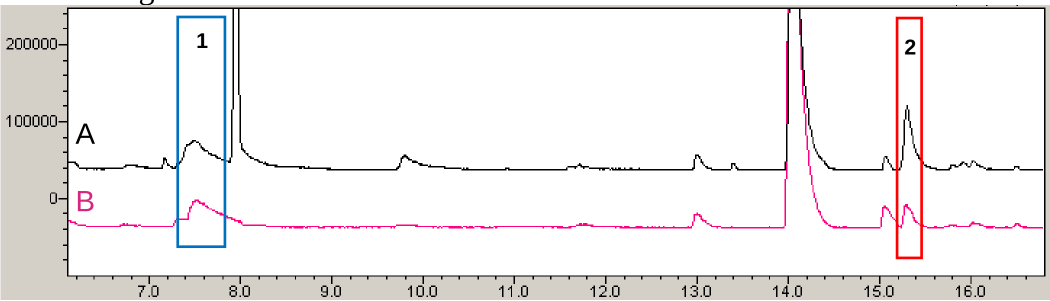

Figure S27. GC-MS spectra of 2,5-dimethylpyrazine (**1**), retention time 7.5 min, produced by *Serratia marcescens* 3B2 cultivated on M9 agar medium supplemented with 0.2% of glucose, 0.5% of L-alanine and 1.5% of non-labelled L-threonine (molecular ion with *m/z* 108) in A and cultivated on the same medium added with L-[U-^{13}C,^{15}N]-threonine (molecular ion with *m/z* 116) in B.

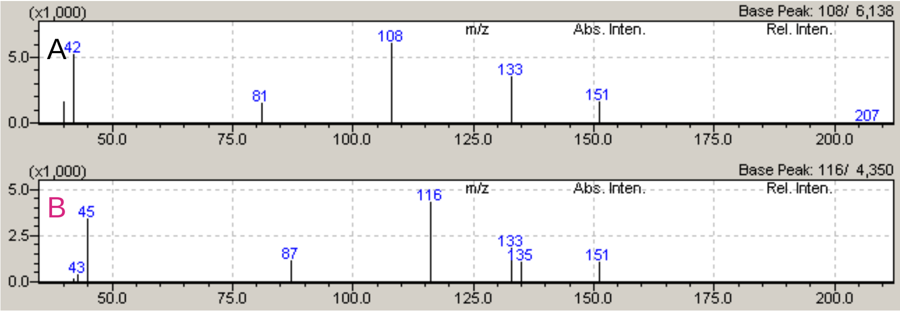

Figure S28. GC-MS spectra of 3-ethyl-2,5-dimethylpyrazine (**2**), retention time 15.4 min, produced by *Serratia marcescens* 3B2 cultivated on M9 agar medium supplemented with 0.2% of glucose, 0.5% of L-alanine and 1.5% of non-labelled L-threonine (molecular ion with *m/z* 136) in A and cultivated on the same medium added with L-[U-^{13}C,^{15}N]-threonine (molecular ion with *m/z* 144) in B.

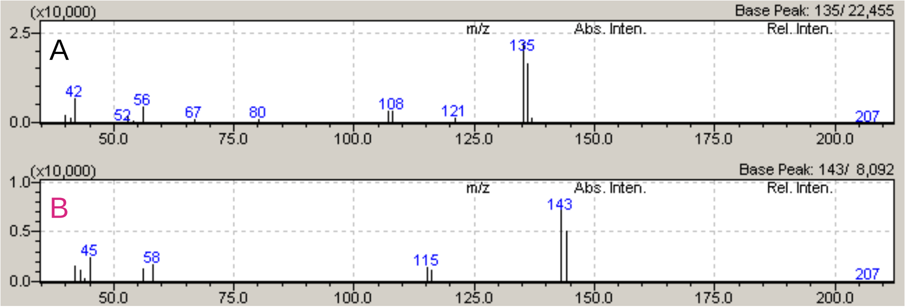

Figure S29. High resolution mass spectra of 2,5-dimethylpyrazine produced by *Serratia marcescens* 3B2 cultivated on M9 agar medium supplemented with 0.2% of glucose, 0.5% of L-alanine and 1.5% of non-labelled L-threonine in A and cultivated on the same medium added with L-[U-^{13}C,^{15}N]-threonine in B. $[M+H]^+$ $C_6H_9N_2$ with *m/z* 109.076, $[M+H]^+$ $C_3{}^{13}C_3H_9N^{15}N$ with *m/z* 113.083 and $[M+H]^+$ $^{13}C_6H_9{}^{15}N_2$ with *m/z* 117.090.

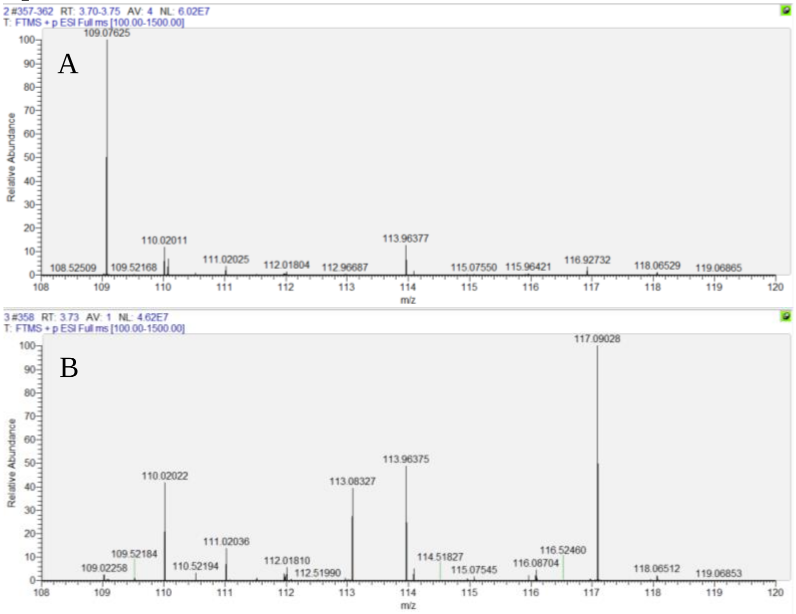

Figure S30. High resolution mass spectra of 3-ethyl-2,5-dimethylpyrazine (**2**) produced by *Serratia marcescens* 3B2 cultivated on M9 agar medium supplemented with 0.2% of glucose, 0.5% of L-alanine and 1.5% of non-labelled L-threonine in A and cultivated on the same medium added with L-[U-^{13}C,^{15}N]-threonine in B. $[M+H]^+$ $C_8H_{13}N_2$ with *m/z* 137.107, $[M+H]^+$ $C_5{}^{13}C_3H_{13}N^{15}N$ with *m/z* 141.114 and $[M+H]^+$ $C_2{}^{13}C_6H_{13}{}^{15}N_2$ with *m/z* 145.121.

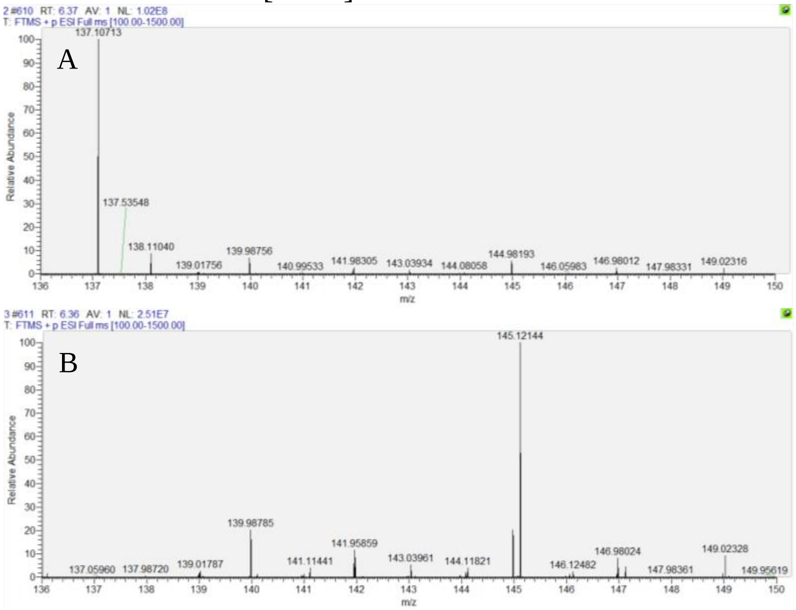

Figure S31. GC-MS analysis of VOCs produced by *Serratia marcescens* 3B2 cultivated on M9 agar medium supplemented with 0.2% of glucose, 1.5% of L-threonine, 0.5% of L-alanine and 0.15% of non-labelled sodium acetate in A and cultivated on the same medium added with 0.15% of U-^{13}C-sodium acetate in B. 2,5-dimethylpyrazine (**1**) peaks are highlighted with blue rectangle and 3-ethyl-2,5-dimethylpyrazine (**2**) with red rectangle.

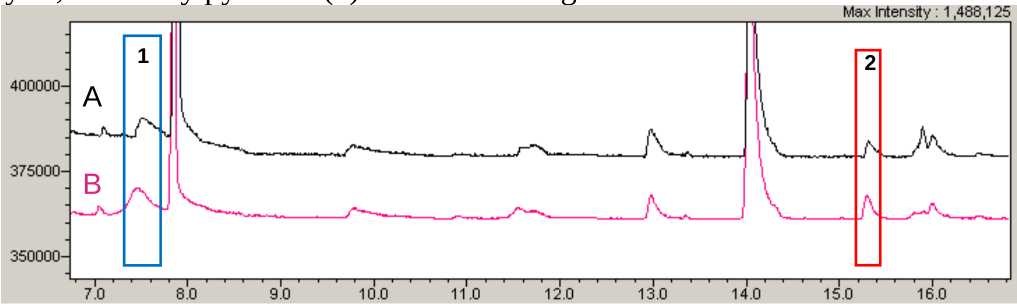

Figure S32. GC-MS spectra of 2,5-dimethylpyrazine (**1**), retention time 7.5 min, produced by *Serratia marcescens* 3B2 cultivated on M9 agar medium supplemented with 0.2% of glucose, 1.5% of L-threonine, 0.5% of L-alanine and 0.15% of non-labelled sodium acetate (molecular ion with *m/z* 108) in A and cultivated on the same medium added with 0.15% of U-^{13}C-sodium acetate (molecular ion with *m/z* 108) in B.

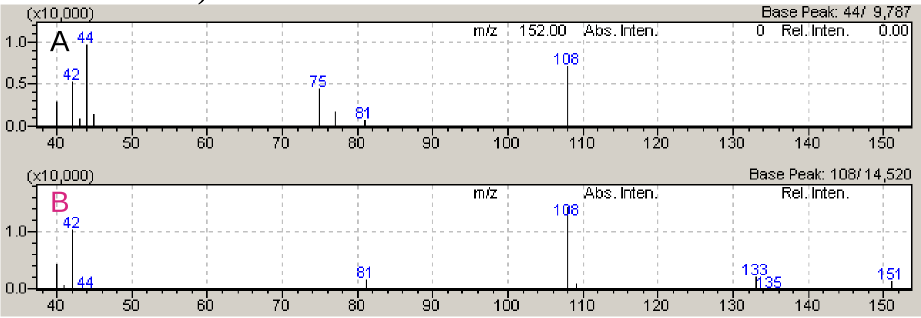

Figure S33. GC-MS spectra of 3-ethyl-2,5-dimethylpyrazine (**2**), retention time 15.4 min, produced by *Serratia marcescens* 3B2 cultivated on M9 agar medium supplemented with 0.2% of glucose, 1.5% of L-threonine, 0.5% of L-alanine and 0.15% of non-labelled sodium acetate (molecular ion with *m/z* 136) in A and cultivated on the same medium added with 0.15% of U-^{13}C-sodium acetate (molecular ion with *m/z* 138) in B.

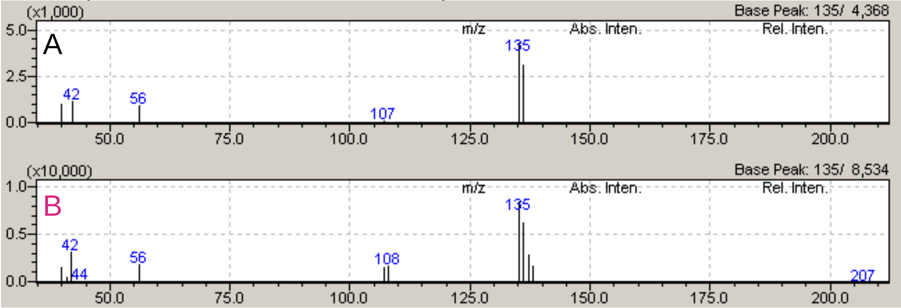

Figure S34. High resolution mass spectra of 2,5-dimethylpyrazine (**1**) produced by *Serratia marcescens* 3B2 cultivated on M9 agar medium supplemented with 0.2% of glucose, 1.5% of L-threonine, 0.5% of L-alanine and 0.15% of non-labelled sodium acetate in A and cultivated on the same medium added with 0.15% of U-^{13}C-sodium acetate in B. $[M+H]^+$ $C_6H_9N_2$ with *m/z* 109.076.

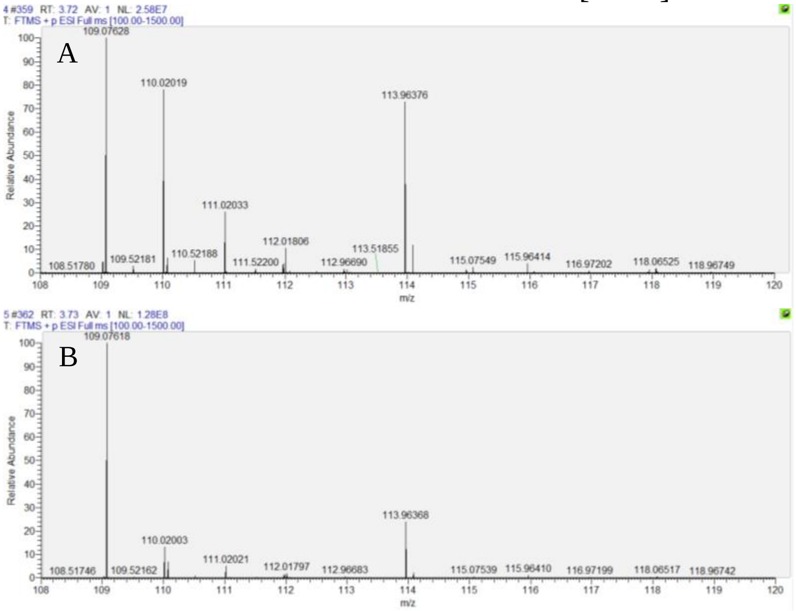

Figure S35. High resolution mass spectra of 3-ethyl-2,5-dimethylpyrazine (**2**) produced by *Serratia marcescens* 3B2 cultivated on M9 agar medium supplemented with 0.2% of glucose, 1.5% of L-threonine, 0.5% of L-alanine and 0.15% of non-labelled sodium acetate in A and cultivated on the same medium added with 0.15% of U-^{13}C-sodium acetate in B. $[M+H]^+$ $C_8H_{13}N_2$ with *m/z* 137.107 and $[M+H]^+$ $C_6{}^{13}C_2H_{13}N_2$ with *m/z* 139.113.

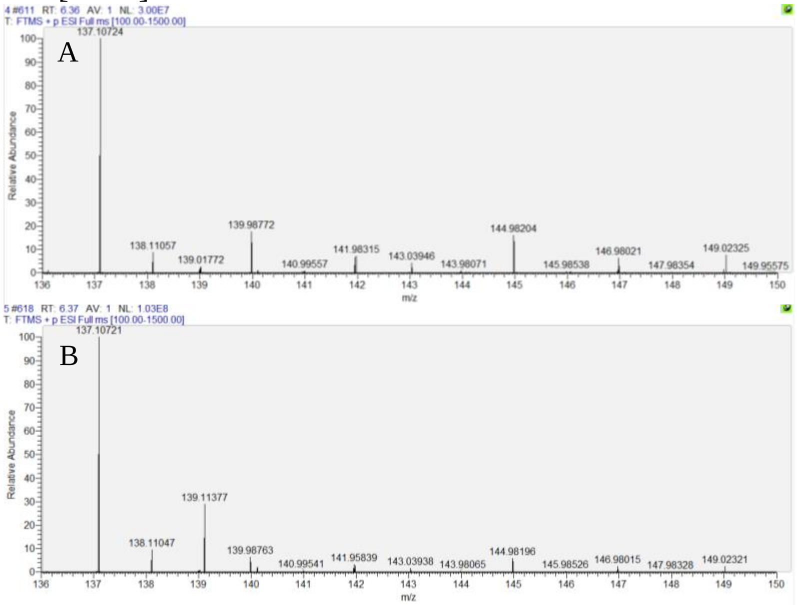

Figure S36. GC-MS analysis of VOCs produced by *Serratia marcescens* 3B2 cultivated on M9 agar medium supplemented with 0.2% of glucose, 1.5% of L-threonine and 0.5% of non-labelled L-alanine in A and cultivated on the same medium added with 0.5% of ^{15}N-L-alanine in B. 2,5-dimethylpyrazine (**1**) peaks are highlighted with blue rectangle and 3-ethyl-2,5-dimethylpyrazine (**2**) with red rectangle.

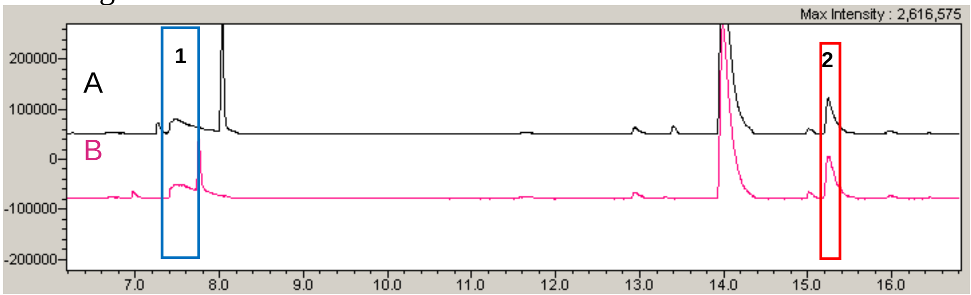

Figure S37. GC-MS spectra of 2,5-dimethylpyrazine (**1**), retention time 7.5 min, produced by *Serratia marcescens* 3B2 cultivated on M9 agar medium supplemented with 0.2% of glucose, 1.5% of L-threonine and 0.5% of non-labelled L-alanine (molecular ion with *m/z* 108) in A and cultivated on the same medium added with 0.5% of ^{15}N-L-alanine (molecular ion with *m/z* 108) in B.

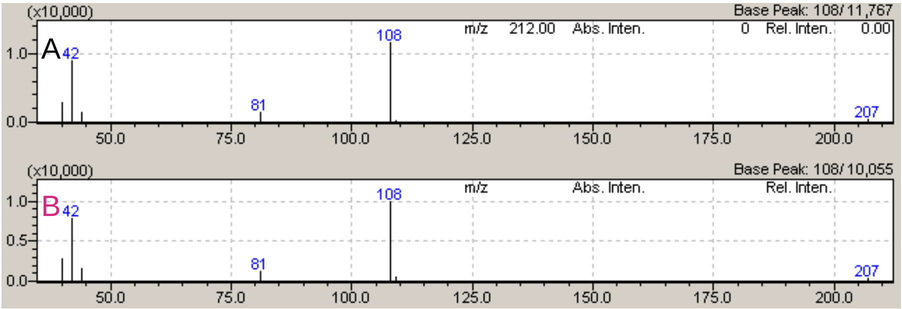

Figure S38. GC-MS spectra of 3-ethyl-2,5-dimethylpyrazine (**2**), retention time 15.4 min, produced by *Serratia marcescens* 3B2 cultivated on M9 agar medium supplemented with 0.2% of glucose, 1.5% of L-threonine, 0.5% of non-labelled L-alanine (molecular ion with *m/z* 136) in A and cultivated on the same medium added with 0.5% of ^{15}N-L-alanine (molecular ion with *m/z* 136) in B.

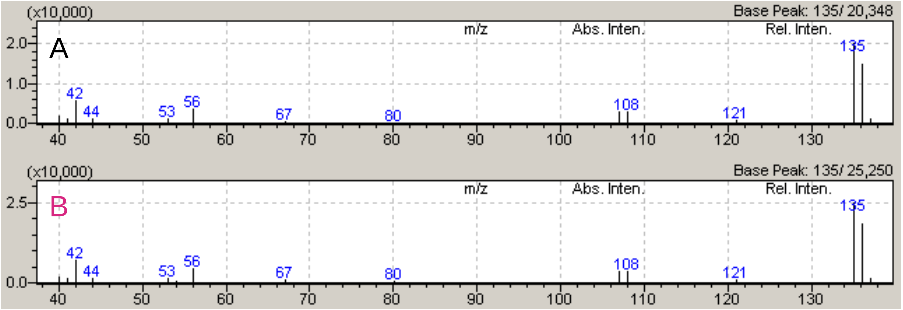

Figure S39. High resolution mass spectra of 2,5-dimethylpyrazine produced by *Serratia marcescens* 3B2 cultivated on M9 agar medium supplemented with 0.2% of glucose, 1.5% of L-threonine and 0.5% of non-labelled L-alanine in A and cultivated on the same medium added with 0.5% of ^{15}N-L-alanine in B. $[M+H]^+$ $C_6H_9N_2$ with *m/z* 109.076.

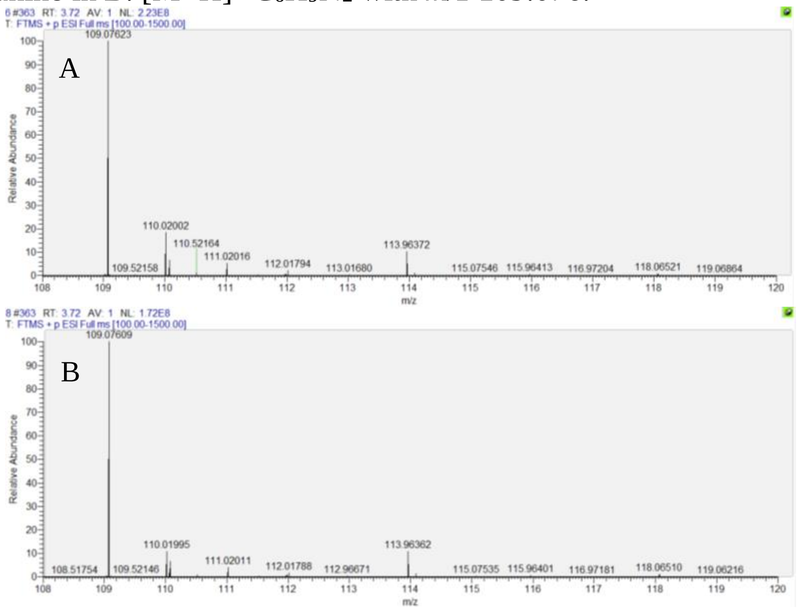

Figure S40. Mass spectra of 3-ethyl-2,5-dimethylpyrazine (**2**) produced by *Serratia marcescens* 3B2 cultivated on M9 agar medium supplemented with 0.2% of glucose, 1.5% of L-threonine, 0.5% of non-labelled L-alanine in A and cultivated on the same medium added with 0.5% of ^{15}N-L-alanine in B. $[M+H]^+$ $C_8H_{13}N_2$ with *m/z* 137.107.

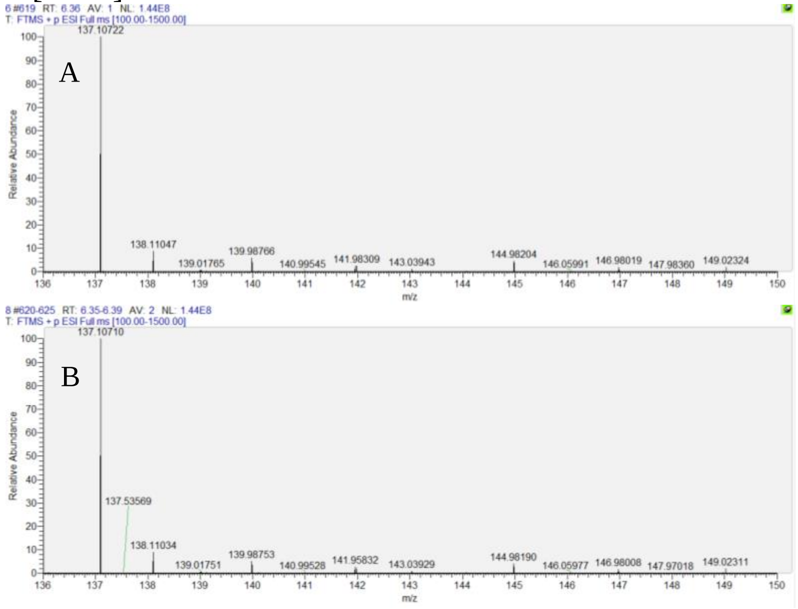

Figure S41. GC-MS analysis of VOCs produced by *Serratia marcescens* 3B2 cultivated on M9 agar medium supplemented with 0.2% of glucose, 1.5% of L-threonine and 0.5% of non-labelled L-alanine in A and cultivated on the same medium added with 0.5% of 3-^{13}C-L-alanine in B. 2,5-dimethylpyrazine (**1**) peaks are highlighted with blue rectangle and 3-ethyl-2,5-dimethylpyrazine (**2**) with red rectangle.

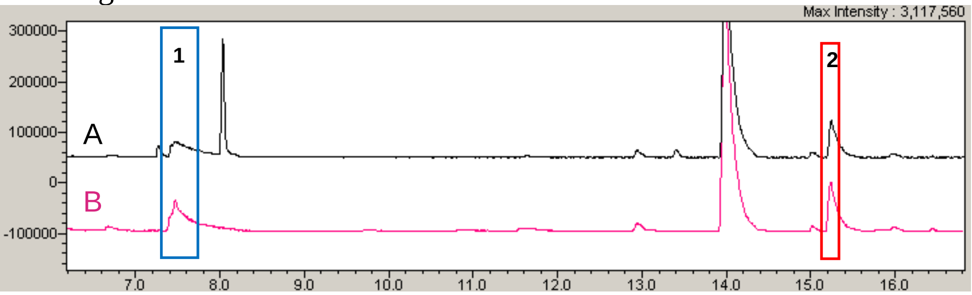

Figure S42. GC-MS spectra of 2,5-dimethylpyrazine (**1**), retention time 7.5 min, produced by *Serratia marcescens* 3B2 cultivated on M9 agar medium supplemented with 0.2% of glucose, 1.5% of L-threonine and 0.5% of non-labelled L-alanine (molecular ion with *m/z* 108) in A and cultivated on the same medium added with 0.5% of 3-^{13}C-L-alanine (molecular ion with *m/z* 108) in B.

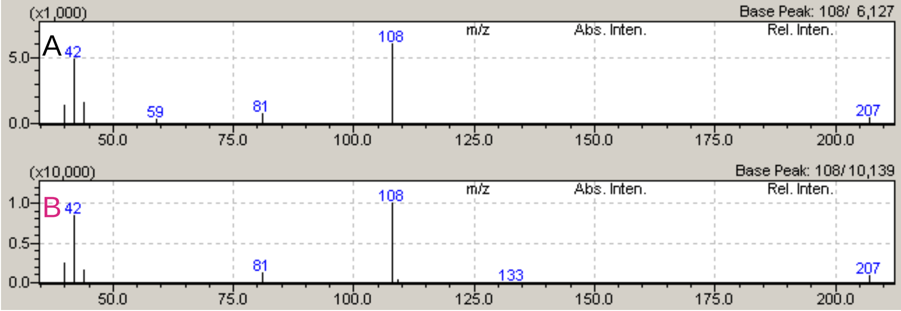

Figure S43. GC-MS spectra of 3-ethyl-2,5-dimethylpyrazine (**2**), retention time 15.4 min, produced by *Serratia marcescens* 3B2 cultivated on M9 agar medium supplemented with 0.2% of glucose, 1.5% of L-threonine, 0.5% of non-labelled L-alanine (molecular ion with *m/z* 136) in A and cultivated on the same medium added with 0.5% of 3-^{13}C-L-alanine (molecular ion with *m/z* 136) in B.

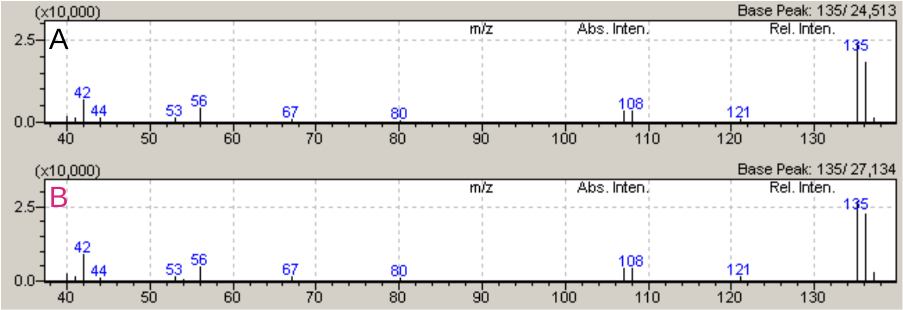

Figure S44. High resolution mass spectra of 2,5-dimethylpyrazine (**1**) produced by *Serratia marcescens* 3B2 cultivated on M9 agar medium supplemented with 0.2% of glucose, 1.5% of L-threonine and 0.5% of non-labelled L-alanine in A and cultivated on the same medium added with 0.5% of 3-^{13}C-L-alanine in B. $[M+H]^+$ $C_6H_9N_2$ with *m/z* 109.076 and $[M+H]^+$ $C_5{}^{13}CH_9N_2$ with *m/z* 110.079.

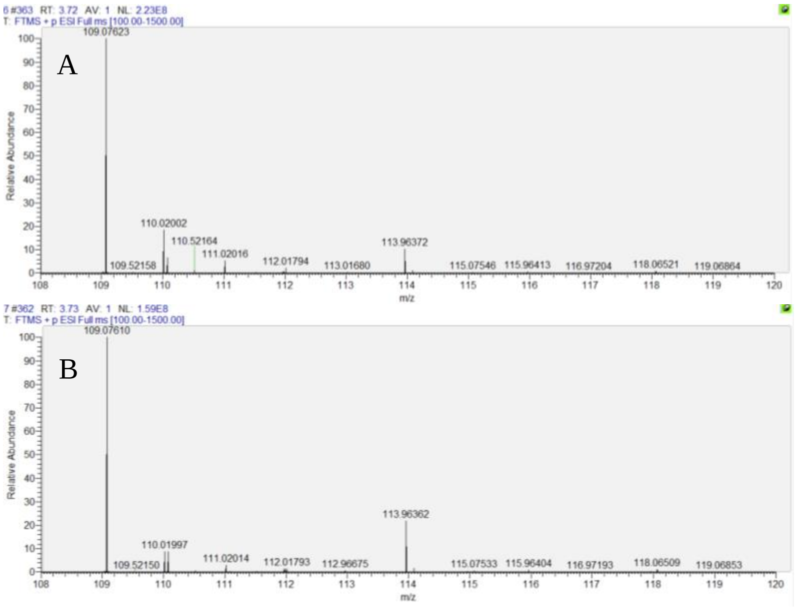

Figure S45. High resolution mass spectra of 3-ethyl-2,5-dimethylpyrazine (**2**) produced by *Serratia marcescens* 3B2 cultivated on M9 agar medium supplemented with 0.2% of glucose, 1.5% of L-threonine, 0.5% of non-labelled L-alanine in A and cultivated on the same medium added with 0.5% of 3-^{13}C-L-alanine in B. $[M+H]^+$ $C_8H_{13}N_2$ with *m/z* 137.107 and $[M+H]^+$ $C_7{}^{13}CH_{13}N_2$ with *m/z* 138.110.

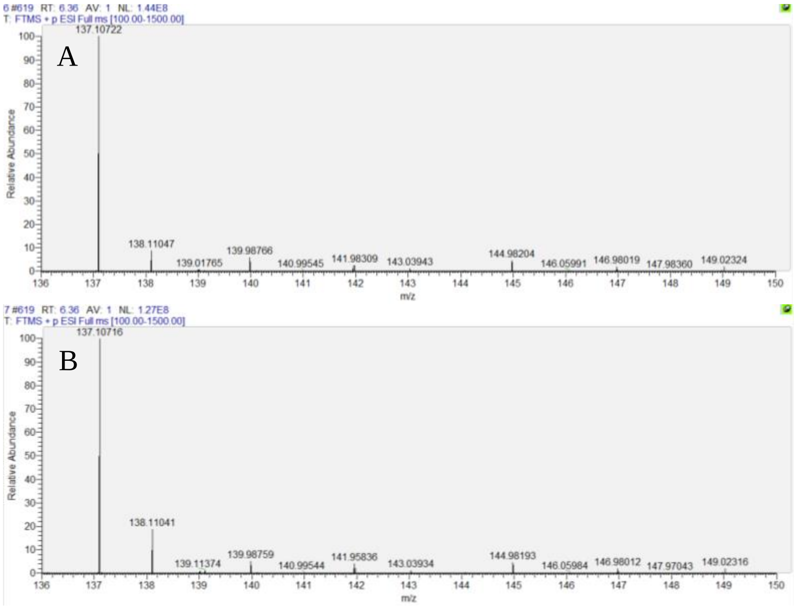

Figure S46. Mass spectra of 2,5-dimethylpyrazine (**1**) produced by *Serratia marcescens* 3B2 cultivated on M9 agar medium supplemented with 0.2% of glucose, 1.5% of L-threonine, 24.4 µM of L-alanine and 24.4 µM of sodium acetate in A, 0.2% of glucose, 1.5% of L-threonine, 24.4 µM of 3-^{13}C-L-alanine and 24.4 µM of sodium acetate in B, and 0.2% of glucose, 1.5% of L-threonine, 24.4 µM of L-alanine and 24.4 µM of U-^{13}C-sodium acetate in C. $[M+H]^+$ $C_6H_9N_2$ with *m/z* 109.076 and $[M+H]^+$ $C_5{}^{13}CH_9N_2$ with *m/z* 110.079.

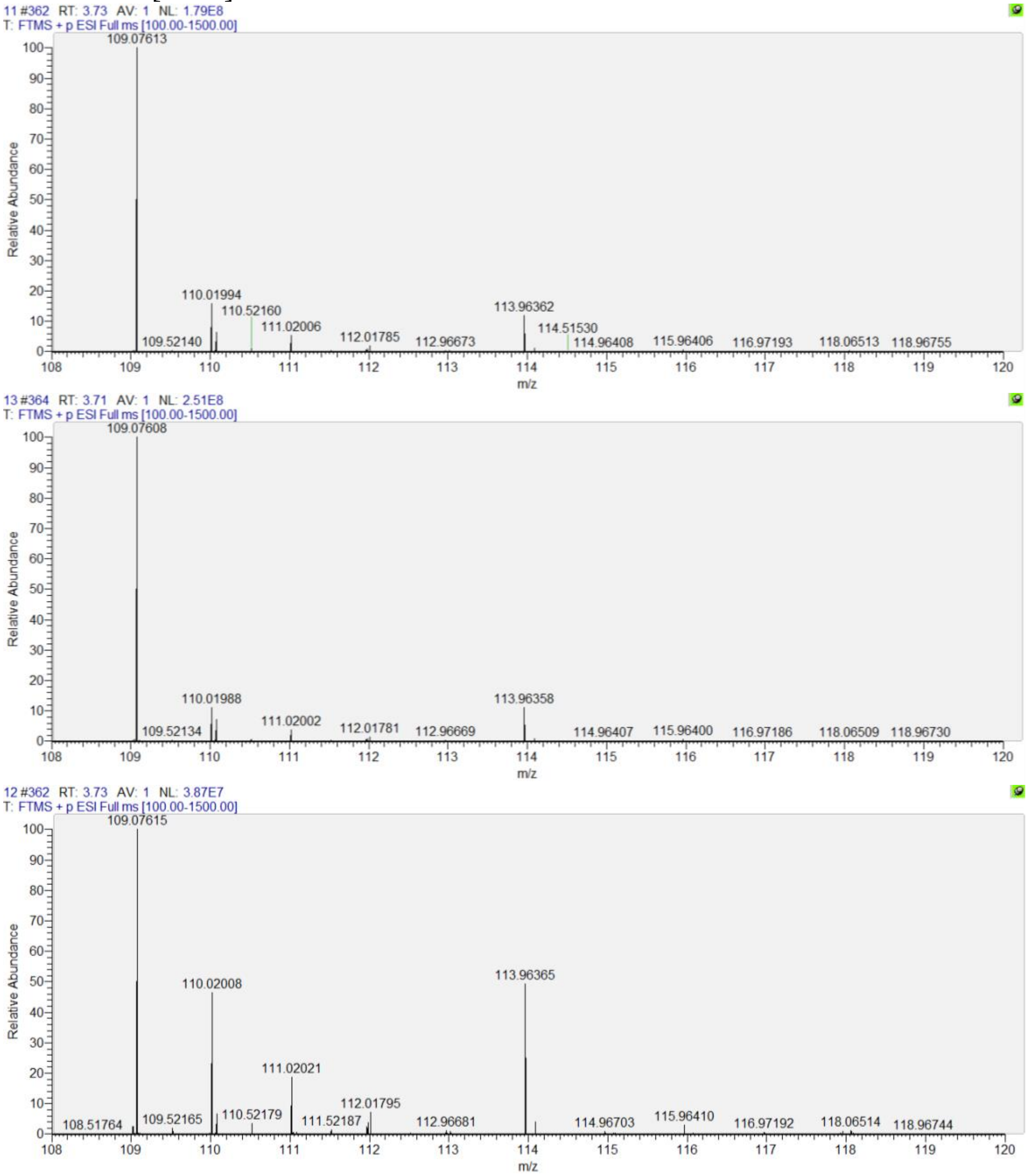

Figure S47. Mass spectra of 3-ethyl-2,5-dimethylpyrazine (**2**) produced by *Serratia marcescens* 3B2 cultivated on M9 agar medium supplemented with 0.2% of glucose, 1.5% of L-threonine, 24.4 µM of L-alanine and 24.4 µM of sodium acetate in A, 0.2% of glucose, 1.5% of L-threonine, 24.4 µM of 3-^{13}C-L-alanine and 24.4 µM of sodium acetate in B, and 0.2% of glucose, 1.5% of L-threonine, 24.4 µM of L-alanine and 24.4 µM of U-^{13}C-sodium acetate in C. $[M+H]^+$ $C_8H_{13}N_2$ with *m/z* 137.107, $[M+H]^+$ $C_7{}^{13}CH_{13}N_2$ with *m/z* 138.110 and $[M+H]^+$ $C_6{}^{13}C_2H_{13}N_2$ with *m/z* 139.113.

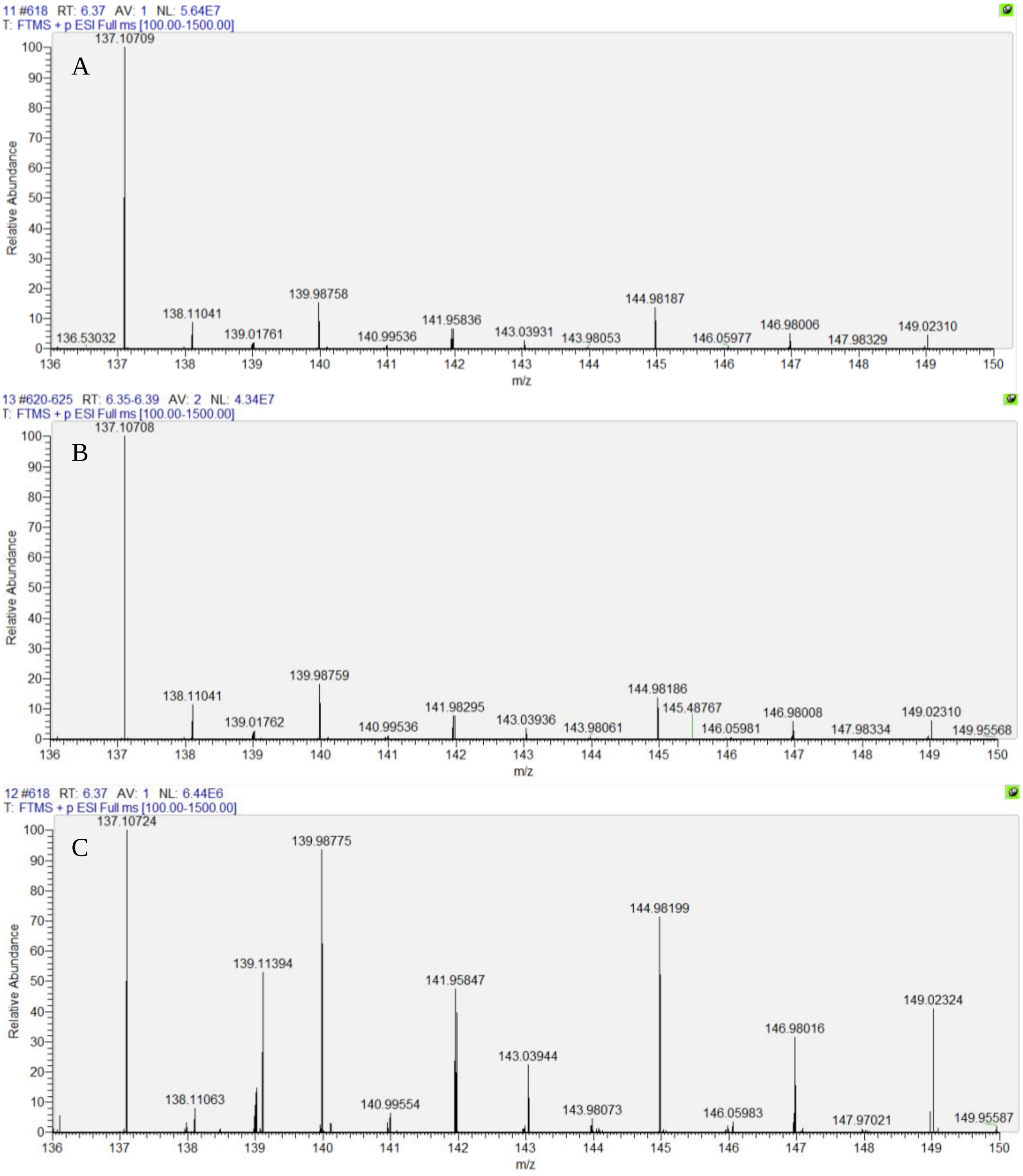

Figure S48. GC-MS spectra of pyrazine **4** produced by *Serratia marcescens* 3B2 cultivated on M9 agar medium supplemented with 0.2% of glucose, 1.5% of L-threonine, 0.5% of non-labelled L-alanine in A, cultivated on the same medium added with 0.5% of ^{15}N-L-alanine in B, added with 0.5% of 3-^{13}C-L-alanine in C, and added with 1.5% of L-[U-^{13}C,^{15}N]-threonine in D.

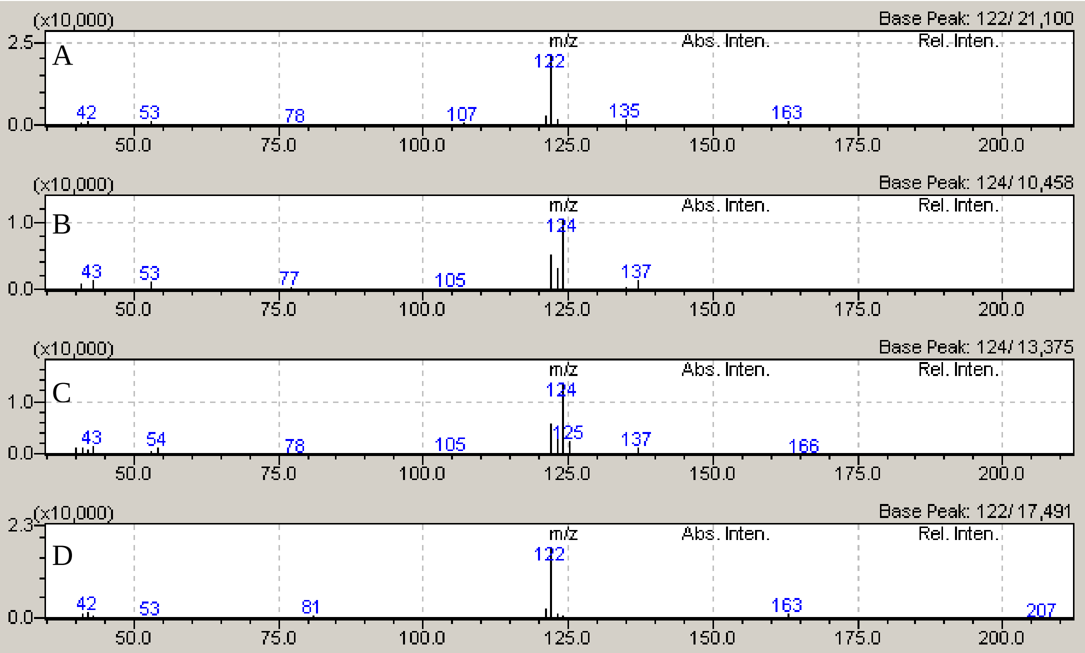

Figure S49. GC-MS spectra of pyrazine **5** produced by *Serratia marcescens* 3B2 cultivated on M9 agar medium supplemented with 0.2% of glucose, 1.5% of L-threonine, 0.5% of non-labelled L-alanine in A, cultivated on the same medium added with 0.5% of ^{15}N-L-alanine in B, added with 0.5% of 3-^{13}C-L-alanine in C, and added with 1.5% of L-[U-^{13}C,^{15}N]-threonine in D.

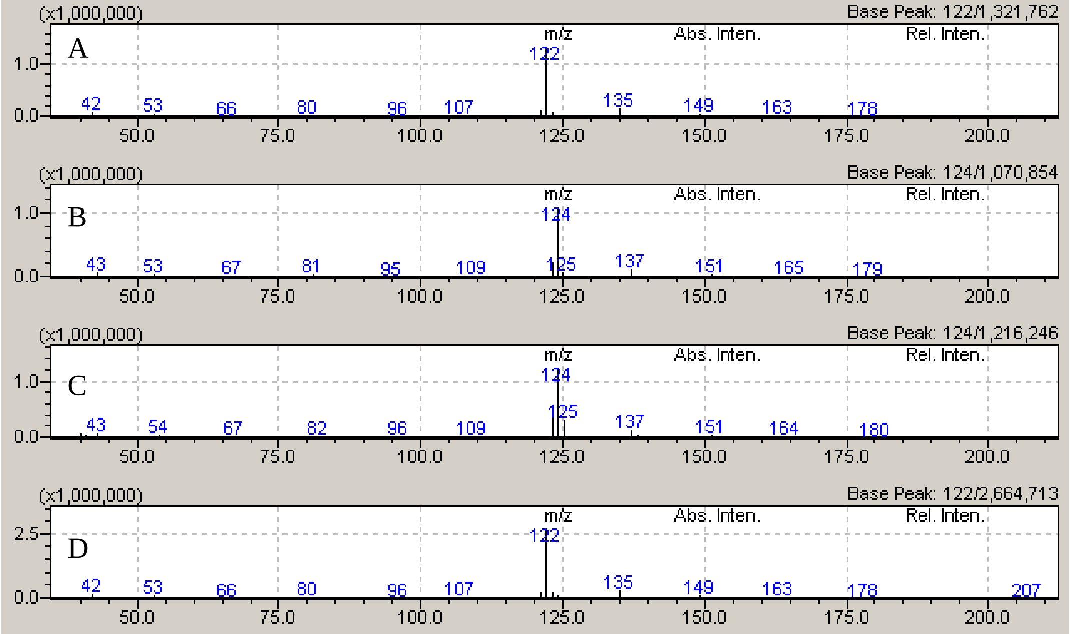

Figure S50. GC-MS spectra of pyrazine **6** produced by *Serratia marcescens* 3B2 cultivated on M9 agar medium supplemented with 0.2% of glucose, 1.5% of L-threonine, 0.5% of non-labelled L-alanine in A, cultivated on the same medium added with 0.5% of ^{15}N-L-alanine in B, added with 0.5% of 3-^{13}C-L-alanine in C, and added with 1.5% of L-[U-^{13}C,^{15}N]-threonine in D.

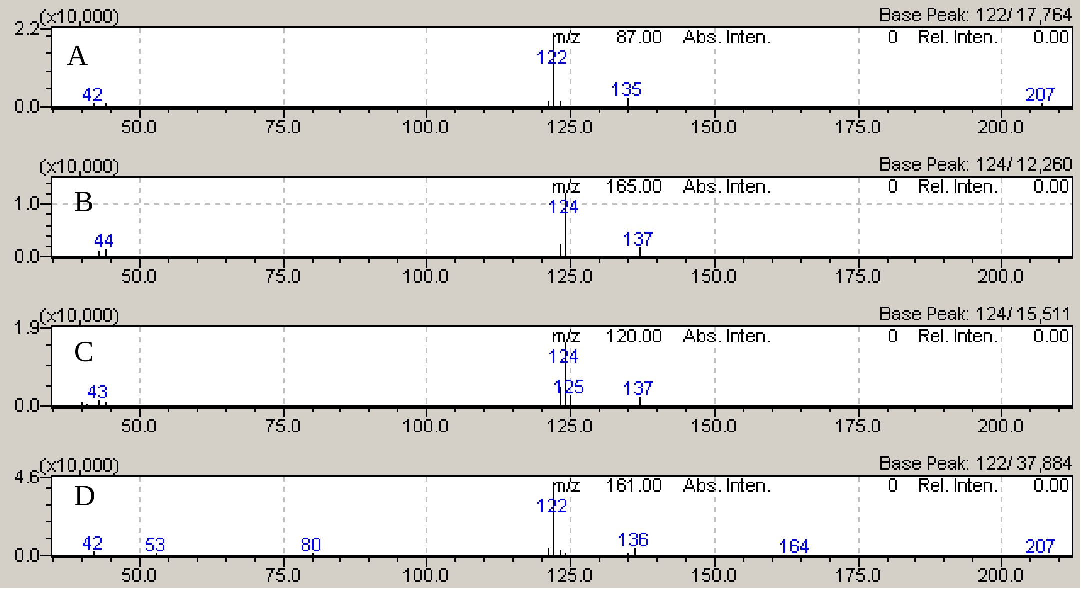

Figure S51. GC-MS spectra of pyrazine **3** produced by *Serratia marcescens* 3B2 cultivated on M9 agar medium supplemented with 0.2% of glucose, 1.5% of L-threonine, 0.5% of non-labelled L-alanine in A, cultivated on the same medium added with 0.5% of ^{15}N-L-alanine in B, added with 0.5% of 3-^{13}C-L-alanine in C, and added with 1.5% of L-[U-^{13}C,^{15}N]-threonine in D.

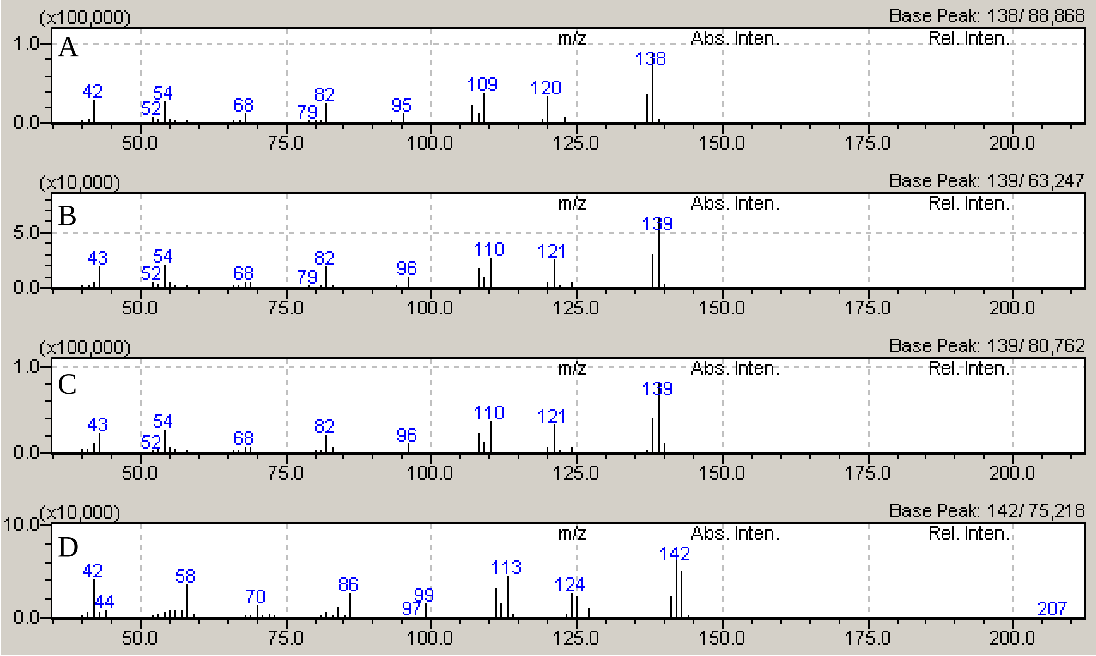

Table S2. Relative ions intensities of protonated pyrazine **1** isotopes on UHPLC-ESI-HRMS spectra of isotope feeding experiments.

	**Ion intensities (%)**			
	$C_6H_9N_2$	$C_5{}^{13}CH_9N_2$	$C_4{}^{13}C_2H_9N_2$	$^{13}C_6H_9{}^{15}N_2$
**L-threonine**	100.00	6.84	0	0
**L-[U-^{13}C,^{15}N]-threonine**	0	0	0	100.00
**sodium acetate**	100.00	6.58	0	0
**U-^{13}C-sodium acetate**	100.00	6.94	0	0
**L-alanine**	100.00	6.73	0	0
**3-^{13}C-L-alanine**	100.00	8.66	0	0
**^{15}N-L-alanine**	100.00	6.88	0	0
**24.4 µM of L-alanine and 24.4 µM of sodium acetate**	100.00	6.60	0	0
**24.4 µM of 3-^{13}C-L-alanine and 24.4 µM of sodium acetate**	100.00	7.44	0	0
**24.4 µM of L-alanine and 24.4 µM of U-^{13}C-sodium acetate**	100.00	6.61	0	0

Table S3. Relative ions intensities of protonated pyrazine **2** isotopes on UHPLC-ESI-HRMS spectra of isotope feeding experiments.

	Ion intensities (%)			
	$C_8H_{13}N_2$	$C_7{}^{13}CH_{13}N_2$	$C_6{}^{13}C_2H_{13}N_2$	$C_2{}^{13}C_6H_{13}{}^{15}N_2$
**L-threonine**	100.00	8.85	0	0
**L-[U-^{13}C,^{15}N]-threonine**	0	0	0	100.00
**sodium acetate**	100.00	8.79	0	0
**U-^{13}C-sodium acetate**	100.00	9.61	29.36	0
**L-alanine**	100.00	8.77	0	0
**3-^{13}C-L-alanine**	100.00	18.67	0	0
**^{15}N-L-alanine**	100.00	8.93	0	0
**24.4 µM of L-alanine and 24.4 µM of sodium acetate**	100.00	8.67	0	0
**24.4 µM of 3-^{13}C-L-alanine and 24.4 µM of sodium acetate**	100.00	11.40	0	0
**24.4 µM of L-alanine and 24.4 µM of U-^{13}C-sodium acetate**	100.00	8.24	52.99	0
